# Mini-dose methotrexate combined with methylprednisolone for the initial treatment of acute GVHD: a multicentre, randomized trial

**DOI:** 10.1186/s12916-024-03395-y

**Published:** 2024-04-25

**Authors:** Yu Wang, Qi-Fa Liu, De-Pei Wu, Zheng-Li Xu, Ting-Ting Han, Yu-Qian Sun, Fen Huang, Zhi-Ping Fan, Na Xu, Feng Chen, Ye Zhao, Yuan Kong, Xiao-Dong Mo, Lan-Ping Xu, Xiao-Hui Zhang, Kai-Yan Liu, Xiao-Jun Huang

**Affiliations:** 1 Peking University People’s Hospital, Peking University Institute of Hematology, National Clinical Research Center for Hematologic Disease, Beijing Key Laboratory of Hematopoietic Stem Cell Transplantation, Collaborative Innovation Center of Hematology, Peking University, Beijing, China; 2 Peking-Tsinghua Center for Life Sciences, Academy for Advanced Interdisciplinary Studies, Peking University, Beijing, China; 3 Department of Hematology, Nanfang Hospital Affiliated to Southern Medical University, Guangzhou, China; 4 Jiangsu Institute of Hematology, The First Affiliated Hospital of Soochow University, Soochow, China; 5 Department of Hematology, Beijing Ludaopei Hematology Hospital, Beijing, China; 6 State Key Laboratory of Natural and Biomimetic Drugs, Peking University, Beijing, China

**Keywords:** Mini-dose, Methotrexate, Acute, GVHD

## Abstract

**Background:**

There is an urgent unmet need for effective initial treatment for acute graft-versus-host disease (aGVHD) adding to the standard first-line therapy with corticosteroids after allogeneic haematopoietic stem cell transplantation (allo-HSCT).

**Methods:**

We performed a multicentre, open-label, randomized, phase 3 study. Eligible patients (aged 15 years or older, had received allo-HSCT for a haematological malignancy, developed aGVHD, and received no previous therapies for aGVHD) were randomly assigned (1:1) to receive either 5 mg/m^2^ MTX on Days 1, 3, or 8 and then combined with corticosteroids or corticosteroids alone weekly.

**Results:**

The primary endpoint was the overall response rate (ORR) on Day 10. A total of 157 patients were randomly assigned to receive either MTX plus corticosteroids (*n* = 78; MTX group) or corticosteroids alone (*n* = 79; control group). The Day 10 ORR was 97% for the MTX group and 81% for the control group (*p* = *.*005). Among patients with mild aGVHD, the Day 10 ORR was 100% for the MTX group and 86% for the control group (*p* = .001). The 1-year estimated failure-free survival was 69% for the MTX group and 41% for the control group (*p* = *.*002). There were no differences in treatment-related adverse events between the two groups.

**Conclusions:**

In conclusion, mini-dose MTX combined with corticosteroids can significantly improve the ORR in patients with aGVHD and is well tolerated, although it did not achieve the prespecified 20% improvement with the addition of MTX.

**Trial registration:**

The trial was registered with clinicaltrials.gov (NCT04960644).

**Supplementary Information:**

The online version contains supplementary material available at 10.1186/s12916-024-03395-y.

## Background

Acute graft-versus-host disease (aGVHD) is the main cause of morbidity and even mortality after allogeneic haematopoietic stem cell transplantation (allo-HSCT) [1]. Corticosteroids remain the standard first-line treatment for aGVHD, although 35–60% of aGVHD patients are refractory to corticosteroid therapy [2–6]. Moreover, there was no significant improvement in the response rate in previous attempts to add other immunosuppressive agents, including anti-interleukin-2 receptor antibody, mycophenolate mofetil (MMF), infliximab, antithymocyte globulin (ATG), or itacitinib, to corticosteroids as first-line aGVHD therapy [7–13]. Therefore, novel front-line therapies targeting nonimmune-related pathways before steroid resistance warrant investigation.

T-cell metabolism plays a role in the pathogenesis of aGvHD. The effect of methotrexate (MTX), a widely used immunosuppressive agent for GVHD prevention and treatment, on metabolic checkpoints has been reported. In the allo-HSCT setting, low-dose (10–15 mg/m^2^) MTX has been used for GVHD prophylaxis since the 1980s. In our previous pilot study, low-dose MTX combined with a low dose of 0.5 mg/kg/d methylprednisolone (MP) as the initial treatment for aGVHD resulted in an overall response in 26 out of 32 aGVHD patients (81%) [14]. However, a correlation was observed between the toxic effects and exposure to low-dose MTX regarding MTX plasma concentrations and intracellular storage in erythrocytes [15]. Hence, recently, a mini dose of 5 mg/m^2^ instead of low-dose MTX was given either for prevention or as salvage treatment for aGVHD, indicating a better safety profile without mitigating efficacy [16, 17].

In everyday practice, a standard dose of 1 or 2 mg/kg/d MP is recommended for the first-line treatment of aGVHD, depending on the initial GVHD grade [1, 6]; additionally, in a haploidentical HSCT setting with earlier onset and poorer outcome of GVHD, treatment of grade I aGVHD is recommended to prevent higher-grade GVHD. Therefore, our group performed a prospective single-centre phase II clinical trial to explore the safety and efficacy of mini-dose MTX (5 mg/m^2^) combined with standard-dose steroids as the initial aGVHD treatment [18]. This combined strategy was well tolerated, showed preliminary efficacy and demonstrated synergistic effects on reducing T-cell alloreactivity in patients with aGVHD. Our preliminary findings provide a rationale for prospective multicentre, randomized trials to validate the efficacy and safety of adding mini-dose MTX to steroids to challenge the standard first-line treatment of systemic steroids for aGVHD.

## Methods

### Study design and subjects

This open-label, randomized, multicentre, phase III trial evaluated the efficacy of mini-dose MTX combined with corticosteroids versus steroids alone for the first-line treatment of acute GVHD. The study was conducted at 4 transplant centres in China (Additional file 1: Table S1). Eligible patients aged 15 to 65 years with acute GVHD (aGVHD) after haematopoietic stem cell transplantation (HSCT) for a haematological malignancy who had evidence of myeloid engraftment and who received no previous treatments for acute GVHD were included. The exclusion criteria included more than one HSCT, life-threatening infection or severe organ dysfunction, and evidence of malignancy relapse or GVHD overlap syndrome. The study protocol was in accordance with the Declaration of Helsinki and was approved by the Institutional Review Board of each participating centre (available in the Additional file 1). All included subjects signed informed consent. This study was registered at http://clinicaltrials.gov/ (NCT 04960644).

### Randomization and masking

Patients were stratified by acute GVHD grade (grade ≤ II or ≥ III) and randomly assigned (1:1) to receive either mini-dose MTX plus corticosteroids (MTX group) or steroids alone (control group) with an interactive web-based response system (IWRS). The computer-generated randomization codes were sent to the IWRS vendor to implement the randomization. Centralized randomization numbers within each stratum were created for treatment assignment, and site staff were instructed to contact the IWRS to obtain the patient identification numbers and initial study drug assignment. The next assignment in the sequence was concealed. The investigators or subjects were not blinded to the assignment. The outcome assessments and data analysis were performed in a blinded pattern.

### Procedures

Donor selection, HLA typing conditioning regimens and GVHD prophylaxis were previously reported in detail [19–21]. All transplant recipients received BUCY-based myeloablative conditioning regimens. Cyclosporine A (CsA), mycophenolate mofetil (MMF), and short-term MTX were given as GVHD prophylaxis to all patients (Table 1). MTX was administered intravenously at 15 mg/m^2^ on Day 1, followed by 10 mg/m^2^ on Days 3 and 6 after matched sibling transplantation or on Days 3, 6, and 11 after haploidentical or unrelated transplantation. Haploidentical and unrelated patients received antithymocyte globulin (ATG) [22] (Table 1). All patients received antibiotic prophylaxis with oral ciproflaxacin, trimethoprim-sulfamethoxazole, posanazole and acyclovir.

**Table 1:** Patient and transplant characteristics (intention-to-treat population)

Patient characteristics	MTX group (*n* = 78)	Control group (*n* = 79)
**Gender**		
Male	42(54%)	42(53%)
Female	36(46%)	37(47%)
**Median age, years, (range)**	34(15–63)	34(15–62)
**MAGIC criteria grade**		
I-II	74(95%)	71(90%)
III-IV	4(5%)	8(10%)
**GVHD risk category**		
Standard	75(96%)	72(91%)
High	3(4%)	7(9%)
**Underlying malignancy**		
Acute myeloid leukemia	32(41%)	41(52%)
Acute lymphoid leukemia	32(41%)	23(29%)
Myelodysplastic syndrome	11(14%)	12(15%)
Other malignant disease	3(4%)	3(4%)
**Graft source**		
Peripheral blood	68(87%)	62(17%)
Bone marrow	10(13%)	17 (21%)
**Donor source**^**a**^		
Matched sibling donor	6(8%)	11(14%)
Matched unrelated donor	4(5%)	6(7%)
Haploidentical donor	68(87%)	62(79%)
**GVHD prophylaxis**^**a**^		
CsA + MMF + MTX	78(100%)	79(100%)
**Baseline organ involvement**		
Specific organ involved		
Skin	71(91%)	65(82%)
Lower gastrointestinal tract or liver	16(21%)	25(31%)
Single or multi organ involvement		
Single	68(87%)	69(87%)
Multi	10(13%)	10(13%)

*GVHD* Graft-versus-host disease, *HLA* Human leukocyte antigen, *MAGIC* Mount Sinai Acute GVHD International Consortium, *CsA* Cyclosporine A, *MMF* Mycophenolate mofetil

^a^The conditioning regimen for haploidentical donor and matched unrelated donor HSCT included cytarabine (4 g/m^2^/day, day –9), busulfan (3.2 mg/kg/day, intravenously days –8 to –6), cyclophosphamide (1.8 g/m2/day, days –5 to –4), semustine (250 mg/m^2^, day –3), and rabbit ATG (thymoglobulin; Imtix Sangstat, Lyon, France, 2.5 mg/kg/day, days –5 to –2). The conditioning regimen for matched sibling donor did not include ATG, otherwise identical to haploidentical donor and matched unrelated donor HSCT. Cyclosporine A (CsA), mycophenolate mofetil (MMF), and short-term MTX were given as GVHD prophylaxis. The dosage of methotrexate was 15 mg/m^2^, administered i.v. on day + 1, followed by 10 mg/m^2^ on days 3, 6, and 11 after haploidentical donor and matched unrelated donor HSCT( 10 mg/m^2^ on days 3,6,after matched sibling HSCT)

**Fig. 1:**
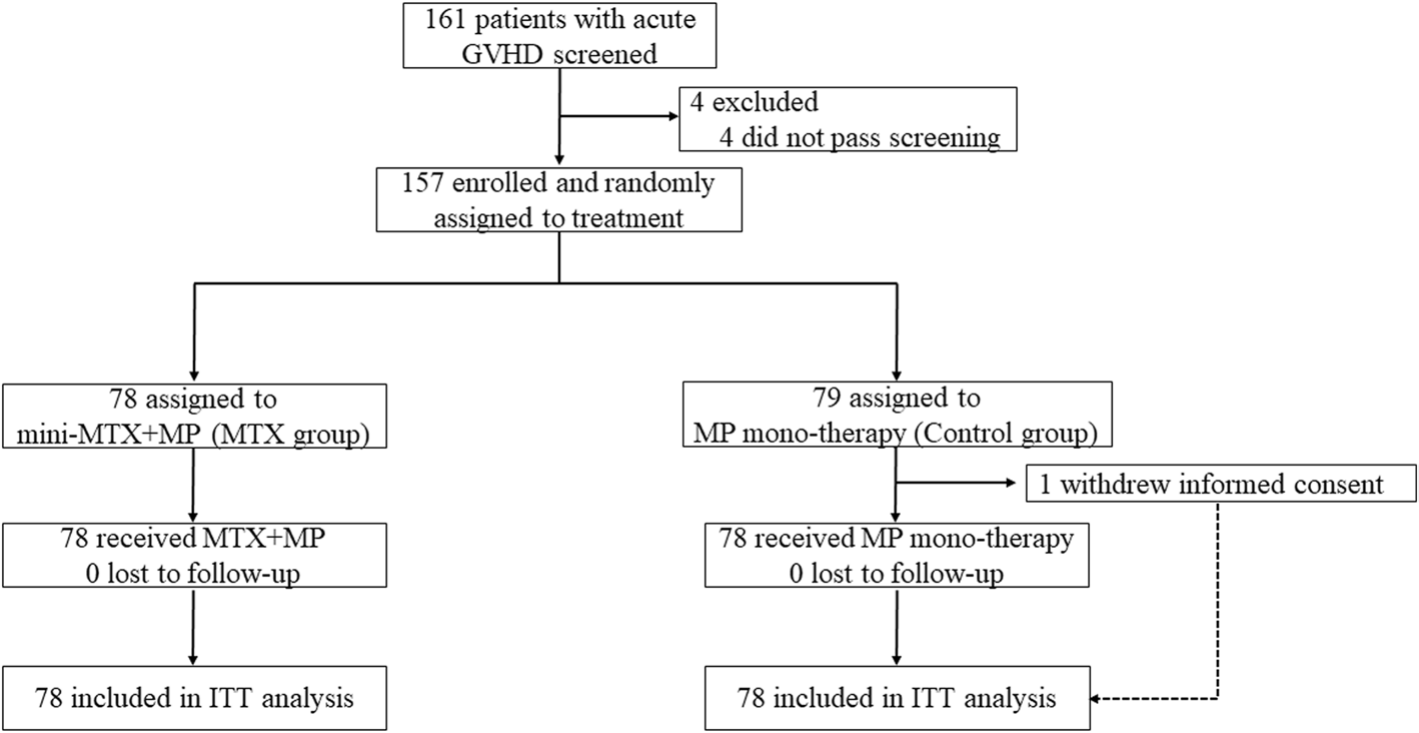
Study profile

Patients assigned to receive MTX received intravenous MTX at a dose of 5 mg/m^2^ and methylprednisolone (MP) at a dose of 1 mg/kg/d according to similar results from an RCT comparing 1 mg/kg/d versus 2 mg/kg/d for Grade I or Grade IIA GVHD [23], and 1 mg/kg/d MP for Grade I-II GVHD did not compromise disease control or mortality and was associated with decreased toxicity compared to 2 mg/kg/d in Mielcarek’s early study [24]. Additionally, in a recent RCT trial using itacitinib, 2 mg/kg/d MP or an appropriate dose for disease severity per institutional guidelines was used [7]. MTX was given on Days 1, 3, 8, and 15 and once every 7 days thereafter. Patients were scheduled to receive at least two doses (number of MTX administrations, Fig. 1) for evaluation of the drug’s efficacy until a complete response (CR) was achieved. Regardless of group assignment, all patients received intravenous 1 mg/kg MP per day (or oral prednisone equivalent). Patients stopped the study treatment at the discretion of the treating investigator, permitted in patients who achieved a complete response, or until withdrawal criteria were met (treatment failure [GVHD progression [25] or partial response requiring additional therapy], unacceptable toxicity, relapse of the underlying malignancy, withdrawn consent, study termination, or if further participation would be injurious to the patient’s health per investigator judgement). The protocol suggested a tapering steroid regimen for both groups (the programmed dose of methylprednisolone (MP) was 1–7 days, 1 mg/kg/d; 8–14 days, 0.8 mg/kg/d; 15–21 days, 0.5 mg/kg/d, and the dose was reduced by half after 5–7 days until it was stopped), but it was not mandated. In general, the taper could not commence sooner than 7 days after randomization.

Patients were observed for 10 days and switched to second-line treatment if there was no response to the initial therapy. Within the observation time of 10 days after initial treatment, second-line treatment was given to patients at 5–7 days with progression of aGVHD or at 10 days with no improvement after initial therapy [25]. The typical sequence of secondary therapy was to add a nonglucocorticoid agent because further escalation of glucocorticoid doses has not been the standard practice at study sites. The second-line treatments followed local institutional practices and included basiliximab at a reported ORR of approximately 80% in steroid refractory GVHD patients in Chinese single-centre large-scale and multicentre real-world studies [26, 27] (Novartis Pharma AG, Basel, Switzerland) at 20 mg/d on Days 1, 3, 8, and weekly afterwards for as long as clinically indicated, ruxolitinib or MMF. According to the updated consensus recommendations of the European Society for Blood and Marrow Transplantation, there is no standard second-line treatment for acute GVHD, and for second-line treatment of acute GVHD, centres should follow their institutional guidelines [1]. For GVHD flares occurring during the corticosteroid taper period, the corticosteroid dose could be re-escalated at the discretion of the investigator and was not considered treatment failure as long as the escalated dose did not exceed 2 mg/kg MP [7].

Patients underwent aGVHD assessments by their treating physicians at screening; every other day for the first 10 days of treatment; at the end of treatment; on Days 14, 28, and 42; every 28 days (range 25–31) during retreatment, if applicable; at safety follow-up (42 days after the end of treatment); and at GVHD follow-up (every 14 days for patients who completed treatment or discontinued early for reasons other than GVHD progression). Prior to initiation of treatment, patients underwent a thorough evaluation to ascertain the severity and extent of their GVHD, including a physical examination, laboratory evaluations and a consultation without tissue biopsy results. Each organ (skin, liver, and gut) was staged 1 through 4 for acute GVHD according to modified criteria based on the schema of the Mount Sinai Acute GVHD International Consortium (MAGIC), and patients were also assigned a grade of acute GVHD (I through IV) based on overall severity [25]. Minnesota GVHD risk status was also evaluated [28]. The time that elapsed between the onset of aGVHD and HSCT was defined as the time from HSCT to the onset of any grade of aGVHD. An independent central adjudication committee verified GVHD diagnosis and grading. The detailed information was recorded in case report forms. A panel of experts determined whether a patient had GVHD and, when present, the grade. Discordance was adjudicated by majority rule.

Moreover, changes in white blood cell count and drug side effects were evaluated to assess drug safety. The Common Terminology Criteria (CTC) for Adverse Events version 4.0 was used to grade the severity of side effects.

### Outcomes

The primary endpoint was the overall response rate (ORR; defined as the proportion of patients with a complete response or partial response) on Day 10 (the study treatment started on Day 1) based on the results of our phase 2 trial ^18^ and according to a joint statement for endpoints for clinical trials testing treatment of aGVHD on repeated occasions rather than daily administration [29]. A complete response (CR) was classified as the complete disappearance of all clinical signs of skin, liver and/or gut GVHD. A partial response (PR) was considered to have occurred if GVHD symptoms in the patient had not completely disappeared but at least one target organ decreased in grade by at least one stage without deterioration of other organs or the emergence of GVHD in other organs. Overall responses (ORs) included CR and PR. No response (NR) or treatment failure was defined as the absence of improvement in any organ involved by aGVHD or worsening in 1 or more organs by 1 or more stages, requiring nonglucocorticoid second-line systemic GVHD therapy.

The secondary endpoints included ORR at Days 28 and 42, duration of response (time from randomization until GVHD progression, flare or death) [7], time to response (interval from treatment initiation to first response), 1-year nonrelapse mortality (NRM, defined as death from causes other than relapse of the underlying malignancy), failure-free survival (FFS, defined as survival without relapse, requirement for additional therapy for acute GVHD, or signs or symptoms of moderate-to-severe chronic GVHD), malignancy relapse rate, overall survival (OS, time from randomization to death from any cause), chronic GVHD incidence (with death and malignancy relapse as competing risks), corticosteroid use, and clinical safety data including incidence of infections (bacterial, fungal, cytomegalovirus (CMV) and Epstein‒Barr virus (EBV) infections).

### Statistical analyses

A sample size of 142 patients was calculated using continuity correction to allow for the detection of an absolute improvement in ORR on Day 10 of 20% (i.e., 90% for MTX vs. 70% for control) with 80% statistical power (one-sided alpha 0.025). The assumed ORR of 70% was based on a grade ≤ II:grade ≥ III ratio of 0.90:0.10 (according to grade ≤ II:grade ≥ III of 8:77 patients in our previous report [30] and approximately 5–15% of high-risk aGVHD in other studies without prior steroids [12, 28, 30, 31]), with stratum-specific response rates of 75% and 30%, respectively [30]. After adjusting for a 10% dropout, the total planned sample size was 156 patients.

The primary endpoint (Day 10 ORR) was assessed via the Cochran–Mantel–Haenszel test with a normal approximation, stratified by GVHD grade. The time course for aGVHD response was estimated using the Kaplan–Meier method. The cumulative incidences of malignancy relapse, NRM, and chronic GVHD were calculated by accounting for competing risks using the Fine and Grey model. For the calculation of nonrelapse mortality, relapse was considered a competing event. The duration of response, OS and FFS were estimated by the Kaplan‒Meier method and compared by the log-rank test. The corresponding hazard ratio (HR) and 95% CI were estimated using the Cox proportional hazards model. All variables in Table 1 were included in the univariable analysis. Only variables with P < 0.10 were included in the multivariable analysis. All reported p values from the primary efficacy analyses are two-sided.

The efficacy-evaluable population included all randomized patients (full analysis set) and was used to summarize baseline characteristics, patient disposition, and analyses of all efficacy data according to the intention-to-treat principle. The population at which safety was evaluated included all patients who received at least one dose of the study drug. The analysis was performed per protocol on August 23, 2022. Endpoints were evaluated from the day of randomization. SPSS 19.0 (Mathsoft, Seattle, WA, USA) and R version 3.4.4 (The R Foundation for Statistical Computing) were used for the data analyses. This trial is registered with ClinicalTrials.gov (NCT04960644).

## Results

### Study population

Between June 25, 2021, and July 12, 2021, 157 patients were enrolled and randomly assigned to receive either mini-MTX plus corticosteroids (*n* = 78) or corticosteroids alone (*n* = 79; Fig. 1). One patient in the control group withdrew informed consent after randomization but was included in the intention-to-treat analysis of efficacy endpoints. Patient demographics and baseline disease characteristics were similar between the two groups (Table 1). Overall, 73 (47%) patients were female, and 147 (94%) were transplanted from haploidentical donors. Eight siblings and two unrelated donor-recipient pairs were fully HLA-matched. At baseline, 12 (8%) of the 157 patients (MTX 4 [5%] of 78; control 8 [10%] of 79) had grade III acute GVHD according to the MAGIC criteria, and 10 (6%; MTX 3 [4%]; control 7 [9%]) had high-risk acute GVHD according to the Minnesota risk stratification (Additional file 1: Table S2).

### Responses

aGVHD occurred at a median of 24 days (range, 10–139) after HSCT. The drugs were administered immediately after aGVHD was diagnosed. On Day 10, 76 (97%, 95% CI 91.0 to 99.6) of the 78 patients in the MTX group and 64 (81%, 71.0 to 88.1) of the 79 patients in the control group achieved an overall response (odds ratio [OR] 8.90, 95% CI, 1.96 to 40.41; *p* = *0.0*05). Among patients with mild (grade I-II) aGVHD, the Day 10 ORR was 100% (95% CI 95.1–100; 74 of 74; 96% [71 of 74] complete response) in the MTX group and 86.1% (75.9–92.1; 61 of 71; 82% [58 of 71] complete response) in the control group (*p* = 0.001). Our post hoc analysis of all participants revealed a significantly increased day-10 complete response rate with MTX (73 [94%] of 78) versus the control (59 [75%] of 79; OR 4.86, 95% CI 1.72‒13.73; *p* = *0.0*03); the other 3 patients in the MTX group and 5 patients in the control group achieved PR by day10. The 3 patients achieving PR by day 10 in the MTX cohort eventually achieved CR after additional doses of MTX at day 12, 14, and 20 after treatment.

**Fig. 2:**
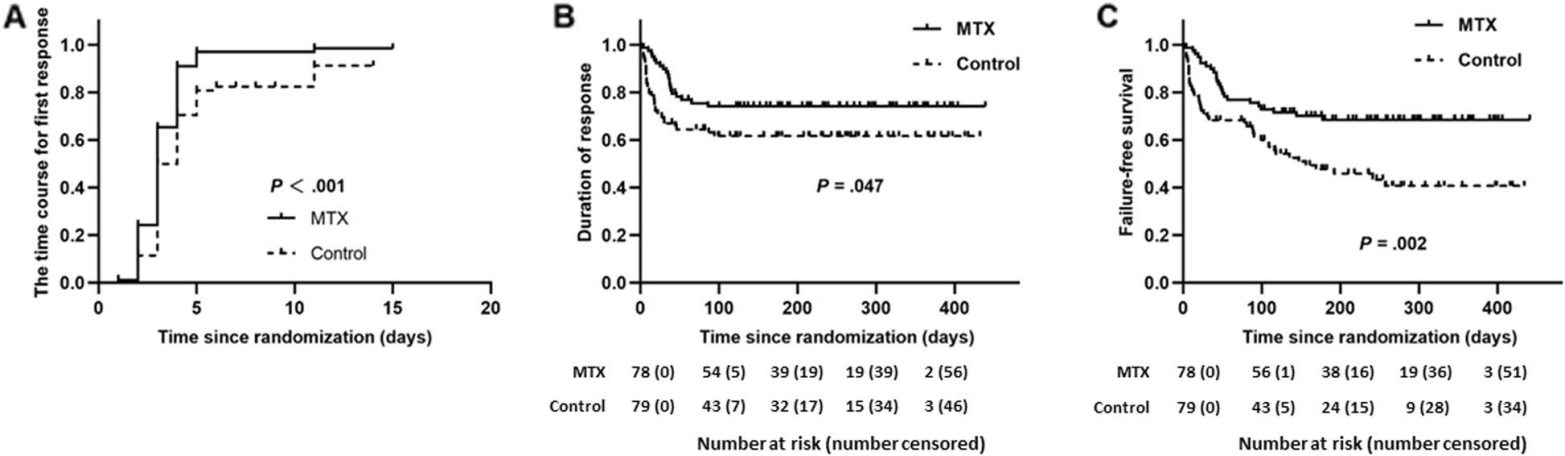
Clinical responses and survival. The time course for the first response (**A**) duration of response (**B**) and failure-free survival (**C**)

Post hoc multivariable analysis revealed that MTX was a significant predictor of response (OR 9.39, 95% CI 1.81 to 48.59; *p* = *0.0*08; Table 2). The Day 10 ORR per aGVHD grade, aGVHD risk and baseline organ involvement are shown in Table 2. An exploratory post hoc subgroup analysis of the Day 10 ORR revealed that MTX treatment favoured the Day 10 ORR compared with the control treatment in almost all subgroups, including age, sex, and transplantation characteristics (Table 2). Since response on Day 7 is normally considered relevant for the decision as to whether a second-line therapy should be started, we also looked at the Day 7 response. The ORRs on Day 7 in the MTX and control groups were 97% (76 of 78 patients) and 81% (64/79, *p* = 0.001), respectively, and the Day 7 CR rates were 74% (58 of 78 patients) and 60% (47/79, *p* = 0.048), respectively. Among the patients who achieved a PR at 7 days after treatment, 15 (19%) and 12 (15%) patients in the MTX and control cohorts, respectively, achieved a CR on Day 10 after treatment without additional second-line treatment. For MTX, the ORR was 92% (72 of 78) on Day 28 and 85% (67 of 78) on Day 42; for the control, these values were 68% (54 of 79) and 65% (52 of 79), respectively (Additional file 1: Table S3). The median time to first response (at least reaching PR) was 3 days for both the MTX-treated group (range 1–11) and the control group (range 2–8); *p* < 0.001, Fig. 2A). The median time needed to achieve a maximal response (CR or PR) was 6 days for both the MTX and control groups. The estimated 6-month response durability was 74% (64 to 84) for the MTX group and 61% (50 to 72) for the control group (*p* = *0.0*47, Fig. 2B).

**Table 2:** Subgroup analyses for overall response at day 10 after treatment

	**MTX cohort (Group A), %(95% CI)**	**Control cohort (Group B), %(95% CI)**	***P* value**
**Diagnosis**			
AML/MDS	97.7(88.2- 99.6)	79.6 (67.1- 88.2)	0.011
ALL or others	97.0 (85.0- 99.4)	84.0 (65.3- 93.6)	0.075
**Patient age**			
< 35	97.5 (87.1- 99.5)	80.4 (65.9- 89.7)	0.029
≥ 35	97.3 (86.5- 99.5)	81.6 (66.5- 90.7)	0.028
**Patient gender**			
Male	100 (91.6- 100)	83.3 (69.3- 91.6)	0.012
Female	94.4 (81.8- 98.4)	78.3 (62.8- 88.6)	0.047
**MAGIC criteria grade**			
I-II	100(95.0- 100)	85.9 (75.9- 92.1)	0.001
III-IV	50.0 (9.1- 90.8)	37.5 (10.2- 74.1)	0.57
**GVHD risk category**			
Standard	100 (95.1- 100)	86.1 (76.2- 92.2)	0.001
High	33.3 (1.7- 87.4)	28.5 (5.1- 69.7)	0.70
**GVHD organ involvement**			
Skin	97.1(90.2- 99.2)	86.1 (75.7- 92.5)	0.026
**Maculopapular rash, < 25% of body surface**	100	92.9	
**Maculopapular rash, 25%-50% of body surface**	93.5	86.7	
**Maculopapular rash, > 50% of body surface**	100	57.1	
Lower GI and/or liver	87.5 (63.9- 96.5)	60.8 (40.7- 77.8)	0.070
**Diarrhea. > 500 but < 1000 mL/d**	100	81.8	
**Diarrhea > .1000 but < 1500 mL/d**	50	50	
**Diarrhea > .1500 mL/d**	0	0	
bilirubin 2-3 mg/dL	-	40	
Bilirubin 3.1–6 mg/dL	100	100	
**Graft source**			
PB	97.1(89.9- 99.1)	80.6 (69.1- 88.5)	0.003
BM	100 (65.5–100)	82.3 (55.8- 95.3)	0.23
**Donor source**			
Matched sibling	100 (39.5–100)	50.0 (13.9- 86.0)	0.091
Haploidentical or unrelated	97.3 (90.6- 99.2)	83.5 (73.4- 90.3)	0.005
**Center**			
Peking university	98.2 (90.8- 99.7)	83.0 (72.1- 90.2)	0.005
Others	95.0 (73.0- 99.7)	71.4 (42.0- 90.4)	0.079

*Abbreviations: OR* Odds ratio, *HR* Hazard ratios, *CI* Confidence interval, *MAGIC* Mount Sinai Acute GVHD International Consortium, *GVHD* Graft-versus-host disease, *AML* Acute myeloid leukemia, *MDS* Myelodysplastic syndrome, *ALL* Acute lymphoid leukemia, *PB* Peripheral blood, *BM* Bone marrow, *GI* Gastrointestinal tract

All of the enrolled patients in the MTX cohort received at least two doses of MTX. MTX was administered at a median of 3 times (range 2–6). Among the 74 patients who achieved CR after MTX treatment, 38 (51%), 4 (5%) and 1 (1%) patients received additional 1, 2, and 3 doses of MTX for consolidation, respectively. In total, 12 patients (15%) in the MTX group and 29 (37%) in the control group received additional nonglucocorticoid GVHD therapy (Additional file 1: Table S4). The most common reasons for second-line therapy were GVHD flares (10 of 12) in the MTX group and GVHD progression (16 of 29) in the control group. Second-line treatment was generally administered later to patients who received MTX than to controls (median time to first dose 32 days for MTX vs. 11 days for control; *p* = *0.0*30). Four patients initially assigned to the steroid-only group subsequently received MTX at doses of 7.5 mg*2, 10 mg*3, 10 mg*2, or 10 mg*3. One of those four patients had no response to MTX and experienced leukaemia relapse afterwards, and the other three patients achieved CR after MTX administration (2 combined with basiliximab).

### Follow-up and survival, cGVHD

The estimated 1-year malignancy relapse rate was similar between the MTX (9%, 2 to 16) and control (7%, 1 to 13) groups (*p* = *0.5*5; Additional file 1: Fig.S1). The estimated 1-year NRM was similar between the MTX (3%, 0 to 6) and control (4%, 0 to 9) groups (*p* = *0.6*4; Additional file 1: Fig. S2A). The primary causes of NRM were infection (*n* = 2) with MTX and infection, GVHD, and organ failure (*n* = 1 each) in the control group (Additional file 1: Table S5).

The 1-year estimates for failure-free survival (defined in the “outcomes” section) were 69% (95% CI 59–79) for the MTX group and 41% (29–53) for the control group (*p* = *0.0*02, Fig. 2C). At a median survival follow-up of 266 days for the MTX group and 261 days for the control group, the estimated 1-year OS was 95% (90 to 99) for the MTX group versus 93% (87 to 99) for the control group (*p* = *0.7*1, Additional file 1: Fig. S2B). Post hoc multivariable analysis of risk factors for FFS showed that MTX was the only protective factor (hazard ratio [HR] 0.49, 95% CI, 0.30 to 0.82; *p* = *0.0*07; Table 3). Severe GVHD was identified as the only adverse factor for OS. Among the 12 patients with severe GVHD, 9 were alive at the last follow-up (5 responded to initial GVHD therapy, and 4 responded to additional GVHD therapy), and the other 3 died (1 died of chronic GVHD at 190 d post-HSCT, 1 died of organ failure at 66 d post-HSCT, and 1 died of malignancy relapse at 221 d post-HSCT).

**Table 3:** Univariable and multivariable analyses for the risk factors of overall response and survival

**Parameters**	**Overall response at day 10 after treatment**		**Overall survival**		**Failure-free survival**	
	**Univariable ** **OR (95%CI)** ***P* value**	**Multivariable** **OR (95%CI)** ***P* value**	**Univariable** **HR (95%CI)** ***P* value**	**Multivariable** **HR (95%CI)** ***P* value**	**Univariable** **HR (95%CI)** ***P* value**	**Multivariable** **HR (95%CI)** ***P* value**
**Gender** female vs male	1.92(0.70–5.24); .20	-	1.30(0.36–4.62); .67	-	0.74(0.46–1.21); .23	-
**Patient age** < 35 vs ≥ 35 years (median)	1.05(0.39–2.82); .90	-	0.40(0.10–1.55); .18	-	1.18(0.72–1.92); .49	-
**MAGIC criteria grade** I-II vs III-IV	17.18(4.67–63.16); * < .001	20.12(4.28–94.37); * < .001	0.18(0.04–0.73); *.016	0.18(0.04–0.73); *.016	0.22(0.11–0.45); * < .001	0.26(0.13–0.52); * < .001
**GVHD risk category** standard vs high	29.06(6.57–128.35); * < .001	-	0.15(0.03–0.58); *.006	-	0.19(0.09–0.40); * < .001	-
**Underlying malignancy** AML/MDS vs ALL or others	0.79(0.281–2.24); .66	-	2.46(0.52–11.61); .25	-	1.20(0.72–2.00); .47	-
**Graft source** PB vs BM	0.96(0.25–3.60); .96	-	1.85(0.23–14.60); .55	-	0.71(0.39–1.29); .26	-
**Donor source**	.71	-	.43	-	.33	-
Matched sibling	0.56(0.14–2.20); .41		0.00 .98		1.45(0.74–2.83); .27	
Unrelated donor	0.00 .99		2.72(0.59–12.72); .20		1.56(0.71–3.41); .26	
Haplo donor	1.0		1.0		1.0	
**Center** Peking university vs others	1.47(0.48–4.46); .49	-	2.61(0.33–20.66); .36	-	0.92(0.52–1.64); .79	-
**Mini-dose MTX treatment** MTX vs control	8.90(1.96–40.41); *.005	9.39(1.81–48.59); *.008	0.78(0.21–2.91); .71	-	0.46(0.27–0.76); * .002	0.49(0.30–0.82); *.007

*Abbreviations: OR* Odds ratio, *HR* Hazard ratios, *CI* Confidence interval, *MAGIC* Mount Sinai Acute GVHD International Consortium, *GVHD* Graft-versus-host disease, *AML* Acute myeloid leukemia, *MDS* Myelodysplastic syndrome, *ALL* Acute lymphoid leukemia, *PB* Peripheral blood, *BM* Bone marrow

^*^P < .05

The median duration of corticosteroid use was similar between the treatment groups (MTX, 55 days; control, 58 days). The median cumulative dose of corticosteroids was also similar between the groups (MTX 1089 mg, control 1151 mg). The estimated 1-year cumulative incidence of any-grade chronic GVHD (cGVHD) was 41% (19 to 53) for the MTX group and 39% (27 to 51) for the control group (*p* = *0.6*9); the estimated 1-year cumulative incidence of moderate-to-severe cGVHD was 12% (4 to 20) for the MTX group and 23% (13 to 33) for the control group (*p* = *0.1*8, Additional file 1: Fig. S3). In other words, the greater FFS in the MTX group than in the control group was attributed to the lower need for second-line therapy and the lower incidence of moderate-to-severe cGVHD.

### Safety, including infection

The population evaluated for safety included 156 patients (MTX, *n* = 78; control, *n* = 78). Adverse events were reported in 71 (91%) of the 78 patients who received MTX and in 66 (85%) of the 78 controls. The most commonly reported adverse events for MTX versus the control were thrombocytopenia or decreased platelet count (27% [21/78] vs. 21% [16/78]), cytomegalovirus viraemia (68% [53/78] vs. 63% [49/78]), and neutropenia or decreased neutrophil count (39% [31/78] vs. 33% [26/78]). Bacteria and/or fungal infections were reported in 11 patients (14%) in the MTX group and 10 patients (13%) in the control group. The specific toxicity of MTX, such as oral mucositis, was also comparable between the groups (Table 4).

**Table 4:** Adverse effects in the safety population

	MTX group (*n* = 78)				Control group (*n* = 78)			
	Grade 1–2	Grade 3	Grade 4	Grade 5	Grade 1–2	Grade 3	Grade 4	Grade 5
**Platelet decreased**^**a**^	11(14%)	6(8%)	4(5%)	0	10(13%)	2(3%)	4(5%)	0
**Neutrophil decreased**^**a**^	18(23%)	13(16%)	0	0	21(27%)	5(6%)	0	0
**Cytomegalovirus infection**	50 (64%)	3 (4%)	0	0	48 (62%)	1(1%)	0	0
**Cardiac**	5(6%)	0	0	0	4(5%)	0	0	1(1%)
**Gastrointestinal**^**b**^	3(4%)	0	0	0	3(4%)	2(3%)	1(1%)	0
**Hepatobiliary/pancreatic disorders**	0	0	0	0	2(3%)	1(1%)	0	0
**Investigations**^**b**^	17(22%)	3(4%)	0	0	18(23%)	4(5%)	1(1%)	1(1%)
**Metabolism and nutrition disorders**	14(18%)	2(3%)	0	0	13(17%)	4(5%)	0	0
**Nervous system disorders**	0	0	0	0	0	0	0	1(1%)
**Renal/genitourinary**	10(13%)	0	0	0	13(17%)	1(1%)	0	1(1%)
**Vascular**	2(3%)	5(6%)	0	0	2(3%)	4(5%)	0	0
**Infections**^**c**^	8(10%)	1(1%)	0	2(3%)	8(10%)	0	0	2(3%)
**Secondary malignancy**^**d**^	-	1(1%)	0	0	-	0	0	0
**mucositis**	16(21%)	2(3%)	0	0	22(28%)	0	0	0

Grade 1–2 adverse events in more than 10% of patients and all grade 3–5 adverse events were recorded

^a^included the patients with platelet and neutrophil counts both decreased

^b^excluded the patients with GVHD

^c^excluded the patients with cytomegalovirus viremia and Epstein Barr virus viremia

^d^secondary malignancy was post-transplant lymphoproliferative disease

Forty-six (59%) patients experienced adverse events related to MTX treatment (vs. 36 [50%] for the control group; Additional file 1: Table S6). Neither the MTX cohort nor the two patients in the control cohort (those with bacterial sepsis) experienced fatal adverse events related to the study treatment.

## Discussion

In a phase 3, multicentre, randomized study, mini-dose MTX plus corticosteroids for the initial treatment of aGVHD significantly improved the ORR (also CR) on Day 10 compared with corticosteroid monotherapy, and adverse events were comparable between the groups, although the prespecified 20% improvement was not achieved with the addition of MTX.

The Day 10 ORR in the MTX group in the current phase III trial (97%) was similar to that in the phase II study^18^. It should be noted that most of the study population had grade I/II aGvHD according to the MAGIC criteria or standard-risk GvHD according to the Minnesota stratification; thus, these patients usually respond well to MP. For Grade I/II aGvHD, the ORR to 1–2 mg/kg MP was reported to be 60%—66% [30, 31], which coincides with our results on Day 7 after treatment in the control group (60%). Additionally, the percentages of enrolled patients with grade ≤ II versus grade ≥ III aGVHD were similar both to those expected (92% compared with a postulated enrolment of 90% per GVHD grade) and to those reported in previous studies (the percentage of enrolled patients with grade ≥ III or high-risk aGVHD was reported to be 5–15% without prior steroids [12, 28, 30, 31]). According to the EBMT guidelines, Grade I patients could have been treated with topical steroids alone [1]. Topical treatment exposure prior to enrolment was not applied in our population. However, in a randomized trial, steroid treatment for acute grade I GvHD prevented progression to grade II GvHD [32], and early onset of GvHD was a substantial negative predictor of survival [23, 28, 32]. The median aGvHD onset time was 24 days in the current population, mainly in the haplo-HSCT setting. Therefore, MP treatment may be initiated for grade I aGvHD after haplo-HSCT, as recommended by the Chinese consensus on GvHD [33]. In other words, mini-dose MTX plus steroids may further improve the response rate for mild aGVHD patients, especially in haploidentical settings. For high-risk or higher-grade GVHD (one important finding from the Mielcarek [23] study was that patients randomized to 1 mg/kg had a markedly greater rate of need for second-line therapy for Grade IIB or higher acute GVHD), 2 mg/kg MP [1, 23] and more accurate prognostic models [34] might enhance the selection of patients most likely to favour the addition of MTX over corticosteroid monotherapy.

The Day 10 ORR was selected as the primary endpoint based on the quick response with mini-dose MTX plus MP and minimizing GVHD evolution from timely judgement of the first-line treatment response; this was in accordance with the findings of studies of steroids in combination with weekly infliximab [11], although the response was commonly determined at Days 14, 28, and 56 after prednisone treatment was initiated [7–10, 28]. According to a joint statement for endpoints for clinical trials testing treatment of aGVHD [29] and based on the results of our phase 2 trial ^18^, the Day 10 ORR was chosen as a specified time point for MTX use on 2–3 repeated occasions (Day 1, 3, 8, rather than continuously) until it was determined that a response was unlikely. Although response on Day 7 is normally considered relevant for the decision as to whether second-line therapy should be started, 19% and 12% of patients who achieved a PR on Day 7 in the MTX and control groups, respectively, further achieved a CR on Day 10 without additional second-line treatment, which in part validates the rationale for this time point of Day 10 assessment. However, multiple publications have shown that Day 28 response is predictive of long-term outcomes and is a generally accepted endpoint for these trials [35]. Our results coincide with these findings, as the Day 28 ORR was more predictive of the 1-year OS (*p* = 0.002) than was the Day 10 ORR (*p* = 0.091; data not shown). Longer-term analyses from this trial, as well as findings from previous studies of MTX for the treatment of steroid-refractory aGVHD [36, 37] or cGVHD [38] and ongoing trials of MTX as a treatment for higher-grade aGVHD with 2 mg/kg MP plus mini-dose MTX (NCT04958538), will further inform the potential role of MTX in GVHD.

The MTX dose used here (5 mg/m^2^) was selected on the basis of better safety control without abrogating efficacy following doses of 5 mg/m^2^ instead of 10–15 mg/m^2^ MTX either as prophylaxis or salvage therapy for aGVHD [16, 17]. The incidences of cytopenias or mucositis were comparable between the groups, suggesting that the addition of mini-dose MTX to standard steroids did not substantially increase the rate of cytopenias or mucositis in aGVHD patients, even those receiving short-term MTX as GVHD prophylaxis, with possible cumulative toxicity. Furthermore, the equivalent rates of cytopenias between cohorts indicate that the haematological toxicities might have been at least partly due to underlying GVHD or comorbidities.

Previous randomized studies evaluating various agents added to steroids for initial aGVHD therapy showed no benefit over steroid monotherapy [7–13]. Additionally, efforts to use sirolimus or itacitinib monotherapy instead of steroids for low-risk naive aGVHD patients have failed despite fewer adverse effects than steroids [39, 40]. For steroid-sparing or downstream benefits, since the addition of MTX results in a better overall response, one might then expect lower steroid use. Our results were consistent with randomized studies evaluating itacitinib combined with steroids for initial aGVHD therapy, which showed comparable steroid use to steroid monotherapy despite a trend toward a greater ORR with the addition of itacitinib for standard-risk GVHD patients (*p* = 0.082). The MP taper schedule was similar between our cohorts, and an increase in MP was seldom adopted in GVHD flare patients. For mild aGVHD, future strategies possibly include either steroid-sparing methods or rapid de-escalation of steroids by adding another effective agent. Recently, glycolysis inhibitors plus steroids have been shown to cooperatively abrogate aGVHD through the role of T-cell metabolism in aGVHD [41]. Investigating predictive biomarkers [19] and evaluating GVHD pathogenesis to choose the appropriate population are necessary to improve the outcomes of patients with aGVHD.

This study has several limitations. First, the follow-up time was short for the malignancy relapse, NRM, survival and quality of life analyses. Second, the post hoc analysis of d28/d42 ORR and cGVHD rates was exploratory and thus should be performed with caution.

## Conclusions

In conclusion, the improvement in the Day 10 ORR (at least for mild aGVHD) with the addition of mini-dose MTX versus corticosteroid monotherapy reached the prespecified significance level, although it did not meet the prespecified 20% improvement with the addition of MTX. Mini-dose MTX was well tolerated for front-line therapy of aGVHD and might offer advantages in terms of FFS. The combined strategy involving the effect of MTX on T-cell metabolism may offer potential for future pathogenesis-oriented therapeutic strategies for aGVHD patients.

### Supplementary Information

**Additional file 1.** **Table S1:** List of principal investigators per center in the study. **Table S2:** GVHD characteristics for the group. **Table S3:** Response at day 7, 10, 28, 42 after treatment. **Table S4:** Second-line therapies used after MTX or control. **Table S5:** Primary cause of death. **Table S6:** Treatment related adverse effects. **Table S7:** Data sharing statement. **Fig. S1**: Cumulative incidence of malignancy relapse. **Fig. S2:** Non-relapse mortality and survival. **Fig. S3:** Cumulative incidence of total chronic GVHD (**A**) and moderate to severe chronic GVHD (**B**).

## Data Availability

The datasets used and/or analysed during the current study are available from the corresponding author on reasonable request.

## Abbreviations

aGVHD: Acute graft-versus-host disease
allo-HSCT: Allogeneic hematopoietic stem cell transplantation
ATG: Antithymocyteglobulin
CsA: Cyclosporine A
CR: Complete response
CMV: Cytomegalovirus
cGVHD: Chronic graft-versus-host disease
EBV: Epstein-Barr virus
GVHD: Graft-versus-host disease
HSCT: Hematopoietic stem cell transplantation
HR: Hazard ratio
IWRS: Interactive web-based response system
MP: Methylprednisolone
MMF: Mycophenolate mofetil
NRM: Non-relapse-mortality
ORR: Overall response rate
