# Supplementary material for: Mini-dose methotrexate combined with methylprednisolone for the initial treatment of acute GVHD: a multicentre, randomized trial
Source: BMC Med. 2024 Apr 25;22:176. doi: 10.1186/s12916-024-03395-y (PMC11044329; doi:10.1186/s12916-024-03395-y)
Supplement: Supplementary file 1 — Additional file 1. Table S1: List of principal investigators per center in the study. Table S2: GVHD characteristics for the group. Table S3: Response at day 7, 10, 28, 42 after treatment. Table S4: Second-line therapies used after MTX or control. Table S5: Primary cause of death. Table S6: Treatment related adverse effects. Table S7: Data sharing statement. Fig. S1: Cumulative incidence of malignancy relapse. Fig. S2: Non-relapse mortality and survival. Fig. S3: Cumulative incidence of total chronic GVHD (A) and moderate to severe chronic GVHD (B). [file 12916_2024_3395_MOESM1_ESM.docx]

**Mini-dose methotrexate combined with methylprednisolone for the initial treatment of acute GVHD: A multicentre, randomized trial**

Yu Wang^1^**^,^**^*^ MD, Qi-Fa Liu^3^**^,^**^*^ MD, De-Pei Wu^4^ MD, Zheng-Li Xu^1^ MD, Ting-Ting Han^1^ MD, Yu-Qian Sun^1,5^ MD, Fen Huang^3^ MD, Zhiping Fan^3^ MD, Na Xu^3^ MD, Feng Chen^4^ MD, Ye Zhao^4^ MD, Yuan Kong^1^ MD, Xiao-Dong Mo^1^ MD, Lan-Ping Xu^1^ MD, Xiao-Hui Zhang^1^ MD, Kai-Yan Liu^1,5^ MD, Xiao-Jun Huang^1,2,6^**^#^** MD

Affiliations:^1^Peking University People’s Hospital, Peking University Institute of Hematology, National Clinical Research Center for Hematologic Disease, Beijing Key Laboratory of Hematopoietic Stem Cell Transplantation, Collaborative Innovation Center of Hematology, Peking University, Beijing, China; ^2^Peking-Tsinghua Center for Life Sciences, Academy for Advanced Interdisciplinary Studies, Peking University, Beijing, China; ^3^Department of Hematology, Nanfang Hospital Affiliated to Southern Medical University, Guangzhou, China; ^4^Jiangsu Institute of Hematology, The First Affiliated Hospital of Soochow University, Soochow, China; ^5^Department of Hematology, Beijing Ludaopei Hematology Hospital, Beijing, China. ^6^State Key Laboratory of Natural and Biomimetic Drugs, Peking University, Beijing, China

**^*^**Yu Wang and Qi-Fa Liu contributed equally to this work.

**^#^**Correspondence to: Xiao-Jun Huang, Peking University People’s Hospital, Peking University Institute of Hematology, National Clinical Research Center for Hematologic Disease, Beijing Key Laboratory of Hematopoietic Stem Cell Transplantation, Collaborative Innovation Center of Hematology, Peking University, Beijing, China; T: 8610-88326006; F: 8610-88324577; Email: huangxiaojun@bjmu.edu.cn

Content

**Supplemental tables**2

Table S12

Table S23

Table S34

Table S45

Table S56

Table S67

Table S78

**Supplemental figures9**

Figure S19

Figure S210

Figure S311

**Study protocol12**

**Table S1: List of principal investigators per center in the study.**

| **Study site** | **Principal investigator** | **Number of patients enrolled** |
| --- | --- | --- |
| Peking University People’s Hospital | Xiao-Jun Huang | 124 |
| Beijing Ludaopei Hematology Hospital | Kai-Yan Liu | 15 |
| Nanfang Hospital Affiliated to Southern Medical University | Qi-Fa Liu | 16 |
| The First Affiliated Hospital of Soochow University | De-Pei Wu | 3 |

**Table S2. GVHD characteristics for the group**

|  | MTX cohort  N(%) | Control cohort  N(%) | Total |
| --- | --- | --- | --- |
| Total enrolled | 78 | 79 | 157 |
| MAGIC grade of aGVHD |  |  |  |
| I-II | 74(95%) | 71(90%) | 145 (92.3%） |
| III | 4(5%) | 8(10%) | 12 （7.6%） |
| IV | 0 | 0 | 0 |
| Cutaneous involvement at onset |  |  |  |
| No rash | 7(9%) | 14 (17%) | 21 (13%） |
| Maculopapular rash, <25% of body surface | 33(43%) | 28(35%) | 61（39%） |
| Maculopapular rash, 25%-50% of body surface | 31(40%) | 30(38%) | 61 (39%） |
| Maculopapular rash, >50% of body surface | 7(9%) | 7(9%) | 14（9%） |
| Generalized erythroderma with bullae formation and desquamation | 0 | 0 | 0 |
| Lower GI abnormalities at diagnosis |  |  |  |
| No diarrhea or diarrhea <500 mL/d | 63(81%) | 60(76%) | 123 (78%） |
| Diarrhea .>500 but <1000 mL/d | 12(14%) | 11(15%) | 23（15%） |
| Diarrhea >.1000 but <1500 mL/d | 2(3%) | 4(5%) | 6 (4%） |
| Diarrhea >.1500 mL/d | 1(1%) | 4(5%) | 5（3%） |
| Severe abdominal pain with or without ileus, or grossly bloody stool  or stool with frank blood or melena | 0 | 0 | 0 |
| Liver abnormalities at diagnosis, bilirubin, mg/dL  bilirubin, mg/dL |  |  |  |
| <2 | 77(99%) | 73(92%) | 150 (96%） |
| 2-3 | 0 | 5(6%) | 5（3%） |
| 3.1-6 | 1(1%) | 1(1%) | 2(1%） |
| 6.1-15 | 0 | 0 | 0 |
| >15 | 0 | 0 | 0 |

Abbreviations: MAGIC=Mount Sinai Acute GVHD International Consortium. GVHD= graft-versus-host disease. GI= gastrointestinal tract

**Table S3 Response at day 7, 10, 28, 42 after treatment**

|  | MTX cohort  (Group A), % | Control cohort  (Group B), % | *P* value |
| --- | --- | --- | --- |
| Day 7 response |  |  |  |
| ORR | 97.4 | 81.0 | 0.001 |
| CR | 74.4 | 59.5 | 0.048 |
| PR | 23.0 | 5.3 |  |
| MAGIC grade ORR |  |  |  |
| I-II | 100 | 85.9 | 0.001 |
| III-IV | 50.0 | 37.5 | 0.57 |
| Day 10 response |  |  |  |
| ORR | 97.4 | 81.0 | 0.001 |
| CR | 93.6 | 74.7 | 0.003 |
| PR | 2.8 | 5.3 |  |
| MAGIC grade ORR |  |  |  |
| I-II | 100 | 85.9 | 0.001 |
| III-IV | 50.0 | 37.5 | 0.57 |
| Day 28 response |  |  |  |
| ORR | 92.3 | 68.4 | 0.001 |
| CR | 92.3 | 68.4 | 0.001 |
| PR | 0 | 0 |  |
| MAGIC grade ORR |  |  |  |
| I-II | 95.9 | 73.2 | <0.001 |
| III-IV | 25.0 | 25.0 | 0.74 |
| Day 42 response |  |  |  |
| ORR | 85.9 | 65.8 | 0.005 |
| CR | 85.9 | 65.8 | 0.005 |
| PR | 0 | 0 | 0 |
| MAGIC grade ORR |  |  |  |
| I-II | 89.2 | 70.4 | 0.006 |
| III-IV | 25.0 | 25.0 | 0.74 |

**Abbreviations: GVHD= graft-versus-host disease; ORR=overall response rate;CR=complete remission; PR= partial remission**

**Table S4:** **Second-line therapies used after MTX or control**

| **Therapy** | **MTX group(n=78)** | **Control group(n=79)** |
| --- | --- | --- |
| **Total** | **12(15%)** | **29(37%)** |
| **Basiliximab** | **7** | **19** |
| **Ruxolitinib** | **3** | **4** |
| **Basiliximab**+**Ruxolitinib** | **1** | **2** |
| **MTX** | **0** | **1** |
| **Basiliximab**+**MTX** | **0** | **3** |
| **Mycophenolate mofetil** | **1** | **0** |

**Table S5:** **Primary cause of death**

| **Primary causes of death** | **MTX group**  **(n=78)** | **Control group**  **(n=79)** |
| --- | --- | --- |
| **Total** | **4(5.1%)** | **5(6.3%)** |
| **Malignancy relapse** | **2** | **2** |
| **GVHD** | **0** | **1 ( chronic GVHD)** |
| **Infections** | **2** | **1** |
| **Organ failure not related to GVHD or infection** | **0** | **1(multiple organ failure)** |

**Abbreviations: GVHD= graft-versus-host disease;**

**Table S6:** **Treatment related adverse effects.**

|  | **MTX group (n=78)** | | |  | **Control group (n=78)** | | |
| --- | --- | --- | --- | --- | --- | --- | --- |
|  | **Any Grade** | **Grade >=3** | **death** |  | **Any Grade** | **Grade >=3** | **death** |
| **Platelet decreased** | 21(27%) | 10(13%) | 0 |  | 16(21%) | 6(8%) | 0 |
| **Neutrophil decreased** | 31(40%) | 13(16%) | 0 |  | 26(33%) | 5(6%) | 0 |
| **Cytomegalovirus infection** | 21(27%) | 1(1%) | 0 |  | 26(33%) | 1(1%) | 0 |
| **Increased ALT or AST** | 7(9%) | 1(1%) | 0 |  | 10(13%) | 0 | 0 |
| **Hyperglycaemia** | 1(1%) | 1(1%) | 0 |  | 0 | 0 | 0 |
| **Vascular** | 1(1%) | 0 | 0 |  | 2(3%) | 2(3%) | 1(1%) |
| **Hypertension** | 2(3%) | 1(1%) | 0 |  | 1(1%) | 1(1%) | 0 |
| **Thrombotic microangiopathy** | 0 | 0 | 0 |  | 1(1%) | 1(1%) | 1(1%) |
| **Infections ^b^** | 9(11%) | 1(1%) | 0 |  | 10(13%) | 2(3%) | 2(3%) |
| **Secondary malignancy (PTLD)** | 1(1%) | 1(1%) | 0 |  | 0 | 0 | 0 |

**ALT= alanine aminotransferase;AST= aspartate aminotransferase; PTLD= post-transplant lymphoproliferative diseases. ^a^ excluded the patients with GVHD; ^b^ excluded the patients with cytomegalovirus viremia and Epstein Barr virus viremia;**

**Table S7: Data sharing statement.**

| **Will individual participant data be available**  **(including data dictionaries)?** | **Yes** |
| --- | --- |
| **What data in particular will be shared?** | **Individual participant data that underlie the results reported in this article, after de-identification (text, tables, figures, and appendices)** |
| **What other documents will be available?** | **Study protocol** |
| **When will data be available (start and end dates)?** | **Beginning 9 months and ending 36 months following article publication** |
| **With whom?** | **Investigators whose proposed use of the data has been approved by an independent review committee (“learned intermediary”) identified for this purpose** |
| **For what types of analyses?** | **For individual participant data meta-analysis** |
| **By what mechanism will data be made available?** | **Proposals may be submitted up to 36 months following article publication.**  **After 36 months the data will be available in our university’s data warehouse but without investigator support other than deposited metadata**  **Information regarding submitting proposals and accessing data may be found at (link to be provided)** |

**Supplemental figures**

**Figure S1** Cumulative incidence of malignancy relapse


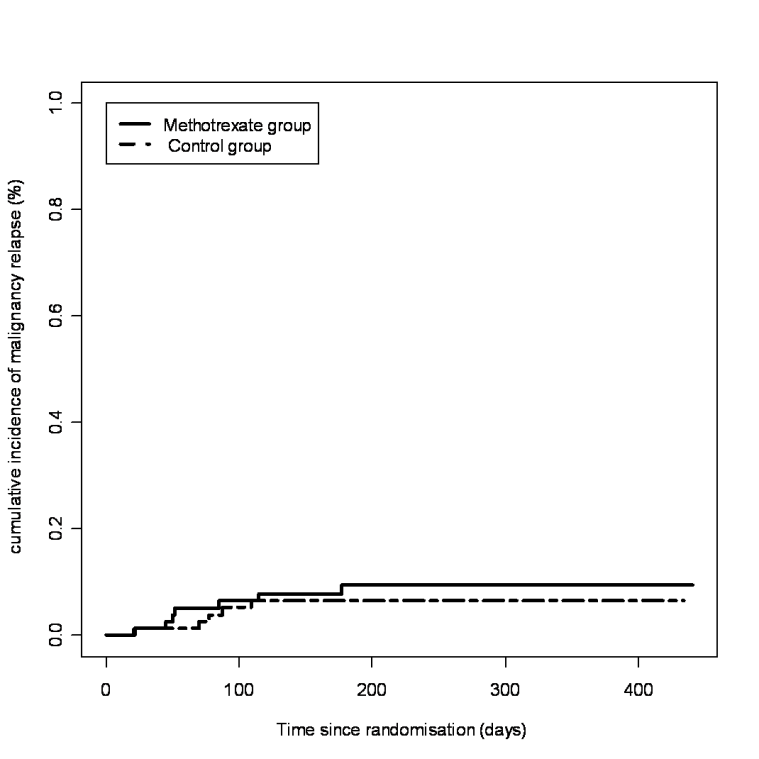


**Figure S2** **Non-relapse mortality and survival**

**
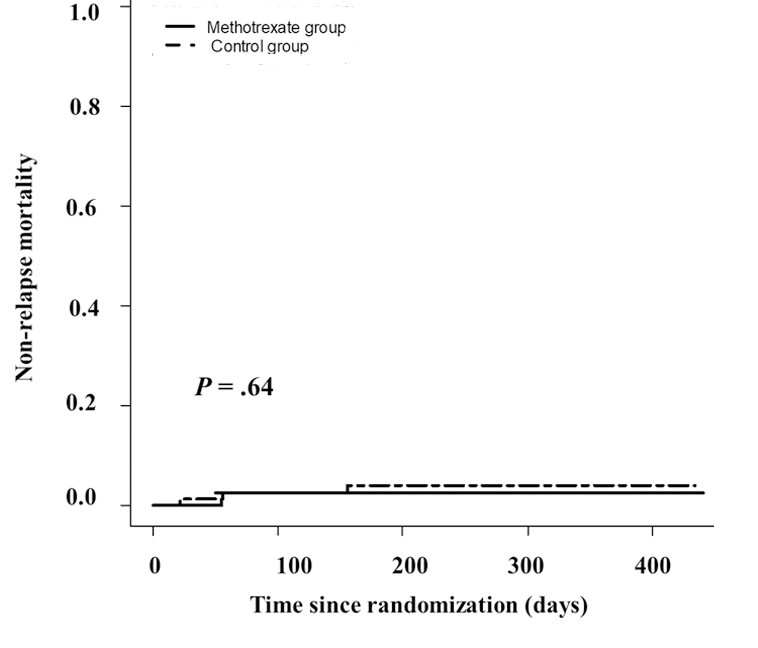

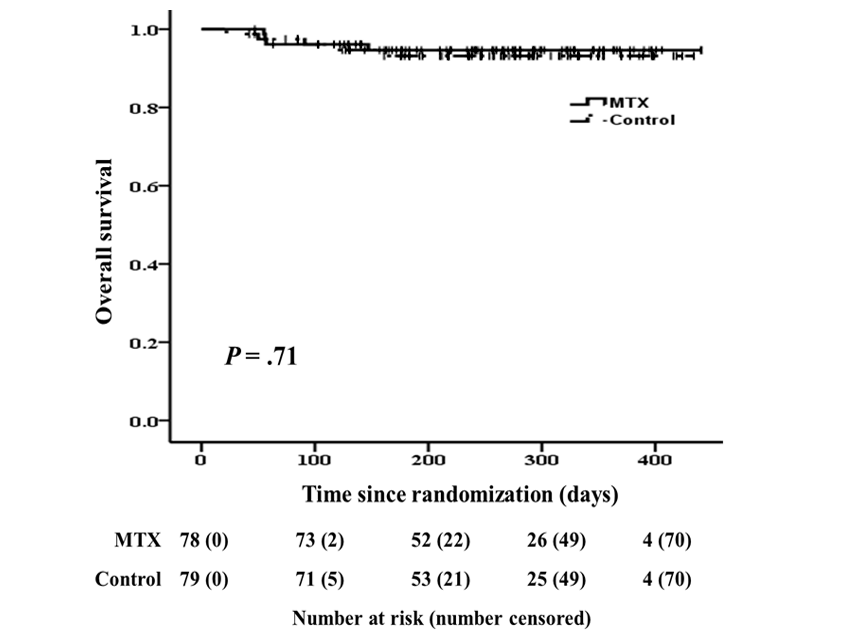
**Cumulative incidence of non-relapse mortality with malignancy relapse as competing risk (A), and overall survival (B).

**Fig S3.** Cumulative incidence of total chronic GVHD (A), and moderate to severe chronic GVHD(B)


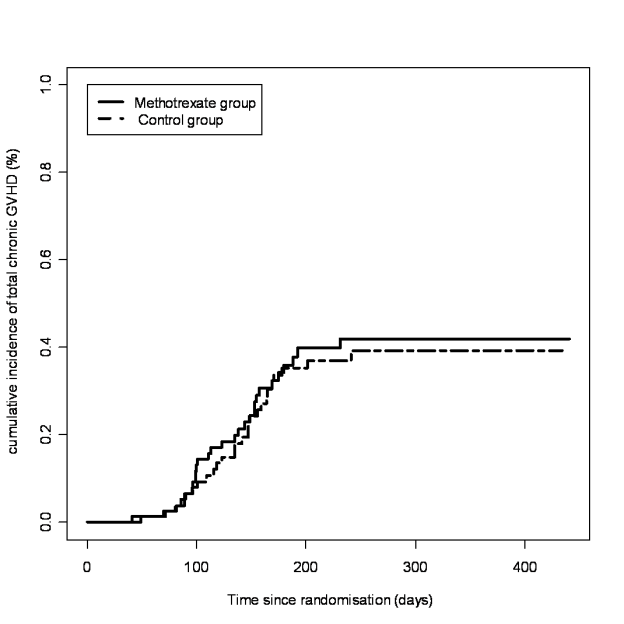

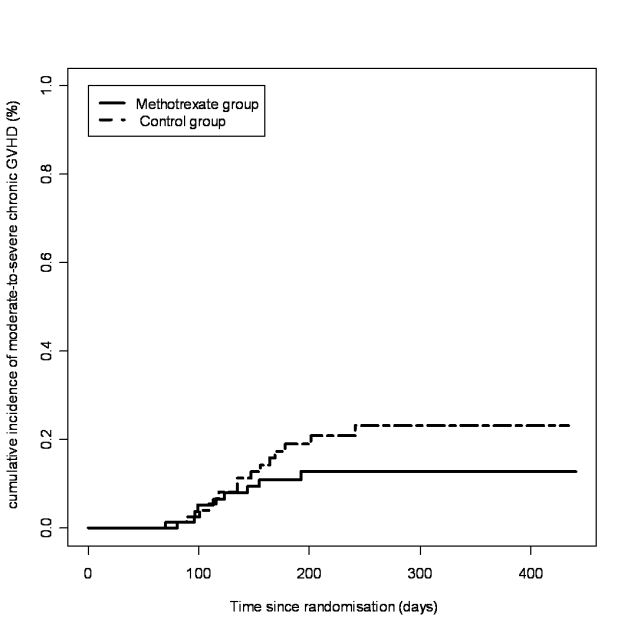


**CLINICAL STUDY PROTOCOL**

**Protocol Title:** **Methotrexate combined with methylprednisolone as a first-line therapy for the treatment of acute graft versus host disease——a Prospective, Multicenter, Randomized, Controlled, Clinical Trial**

**Protocol Number: NCT04960644**

**Study Phase: 3**

**Indication: acute graft versus host disease post allogeneic hematopoietic stem cell transplantation**

**Applicant Institution:** **Peking University People’s Hospital**

**Principal institution: Peking University People’s Hospital**

**Nanfang Hospital, Southern Medical University**

**First Affiliated Hospital of Soochow University**

**Beijing Ludaopei Hematology Hospital**

**Version: 1.0**

**Date: 20210329**

**Remark: the primary version of this protocol was in Chinese. We have translated it into English.**

**INVESTIGATOR'S STATEMENT**

I have received and completely reviewed the following protocol (Protocol Number NCT04960644), including all appendices:

As Principal Investigator, I understand and agree to conduct this clinical study as described and will comply with the ethical and regulatory considerations delineated herein.

**Study Title**

Methotrexate combined with methylprednisolone as a first-line therapy for the treatment of acute graft versus host disease——a Prospective, Multicenter, Randomized, Controlled, Clinical Trial

**Principal Investigator Signature and Contact Information**

**Principal Investigator (print) Xiao-Jun Huang**

**Principal Investigator (signature)
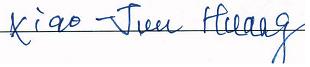
**

**Date of Signature**

**Institution/Affiliation Peking University People’s Hospital**

**City, Province, Country Beijing China**

# Study Synopsis

| **Study title** | Methotrexate combined with methylprednisolone as a first-line therapy for the treatment of acute graft versus host disease——a Prospective, Multicenter, Randomized, Controlled, Clinical Trial |
| --- | --- |
| **Protocol number** | NCT04960644 |
| **Indication** | acute graft versus host disease post allogeneic hematopoietic stem cell transplantation |
| **Study phase** | 3 |
| **Study applicant** | Peking University People’s Hospital |
| **Study center** | Nanfang Hospital, Southern Medical University  Peking University People’s Hospital  First Affiliated Hospital of Soochow University  Beijing Ludaopei Hematology Hospital |
| **Number of subjects planned** | Approximately 156 subjects (78 in MTX+MP group and 78 in control group) will be randomized. |
| **Study duration** | Estimated to be 1 year |
| **Objectives** | **Primary objective:**  Overall response rate (ORR) for aGvHD treatment after treatment  Overall response rate is defined as the proportion of patients demonstrating a complete response or partial response without requirement for additional systemic therapies for an earlier progression, mixed response or non-response.  [Time Frame: 10 days]  **Secondary objectives:**  The secondary endpoints included ORR at days 28, and 42, duration of response (time from randomisation until GVHD progression or death), time to response (interval from treatment initiation to first response), 6-month non-relapse-mortality (NRM, defined as death from causes other than relapse of the underlying malignancy), failure-free survival (FFS, defined as alive without relapse, requirement for additional therapy for acute GVHD, or signs or symptoms of moderate-to-severe chronic GVHD), malignancy relapse rate, overall survival (OS, time from randomisation to death from any cause), chronic GVHD incidence (with death and malignancy relapse as competing risks), corticosteroid use, clinical safety data including incidence of infections. |
| **Study design** | This is a prospective, open-label, randomized, phase 3 study comparing MTX plus corticosteroid with corticosteroid for treating patients with acute graft versus host disease post allo-HSCT.  Approximately 156 subjects will be randomized in a 1:1 ratio to receive MTX plus corticosteroid (78 subjects) or corticosteroid (78 subjects) for treating aGvHD post-transplantation. The computer-generated randomization codes were sent to the IWRS vendor to implement the randomization, and implemented through an interactive web-based response system.  **Study Group:** Patients assigned to MTX received intravenous MTX at a dose of 5 mg/m^2^ and methylprednisolone (MP) at a dose of 1mg/kg/d. MTX was given on days 1, 3, 8, and 15, and once every 7 days afterwards until aGVHD was CR.  **Control Group:** Patients received intravenous MP 1 mg/kg per day (or oral prednisone equivalent). |
| **Inclusion criteria** | Subjects eligible for enrolment in this study must meet all of the following criteria:   1. Aged 15-65 years old; 2. Patients who are fully informed and sign informed consent by themselves or their guardians; 3. Patients receiving allogeneic hematopoietic stem cell transplantation; 4. Patients with acute graft-versus-host disease of grade I-IV were diagnosed after transplantation; 5. Patients with stable engraftment of myeloid and platelets; 6. ECOG score ≤3. |
| **Exclusion criteria** | Subjects meeting any of the following criteria are ineligible for this study:   1. Patients with more than one HSCT; 2. Patients with severe brain, heart, kidney or liver dysfunction unrelated to graft-versus host disease; 3. Patients with uncontrollable active infection; 4. Patients with recurrence of primary hematologic malignant disease; 5. Expected survival is less than 3 months; 6. Patients who have histories of severe allergic reactions; 7. Pregnant or lactating women; 8. The researcher judges that there are other factors that are not suitable for participating. |
| **Study treatment** | **Study: MTX and corticosteroid**  Methylprednisolone 1 mg/kg/day was given for 10 days and then gradually reduce the dose according to patient's response MTX (5mg/m^2/day) was given on days 1, 3, and 8, and repeated weekly until aGvHD was CR.  **Control: corticosteroid**  Methylprednisolone 1 mg/kg/day was given for 10 days and then gradually reduce the dose according to patient's response. |
| **Sample size determination** | This trial was designed to test the hypothesis that MTX plus methylprednisolone was superior to methylprednisolone in the treatment of aGvHD post transplantation. A sample size of 142 patients was calculated using continuity correction to allow for the detection of an absolute improvement in ORR at day 10 of 20% (ie, 90% for MTX vs 70% for control) with 80% statistical power (one-sided alpha 0.025). The assumed ORR of 70% was based on a grade ≤II:grade≥III ratio of 0.90:0.10, with a stratum-specific response rate of 75% and 30%, respectively. After adjusting for a 10% dropout, the total planned sample size was 156 patients. The sample size calculation was performed using PASS software(version 11.0). |
| **Statistical analysis** | Statistical analysis is performed based on the intent-to-treat (ITT) population, which includes all randomized subjects.  **Primary Efficacy Analysis:**  1. Overall response rate (ORR) for aGvHD treatment after treatment  The primary endpoint was the response rate for aGvHD treatment, which was assessed at 10 days post treatment.  **Secondary Efficacy Analysis:**  2. Overall response rate (ORR) for aGvHD treatment at 28 days after treatment  3. Overall response rate (ORR) for aGvHD treatment at 42 days after treatment  4. Number of participants with treatment-related adverse events as assessed by CTCAE v4.0  Data collection including questionnaires at individual and group visits and physician interviews at individual visits will be used to assess participants for treatment-related adverse events.  [Time Frame: 42 days]  5. cGvHD  The cumulative incidence of chronic GVHD  [Time Frame: 1 years]  6. Infection  The cumulative incidence of severe infection  [Time Frame: 1 year]  7. Relapse  The cumulative incidence of relapse  [Time Frame: 1 year]  8. Non-relapse mortality  The cumulative incidence of non-relapse mortality  [Time Frame: 1 year]  9. Overall survival  The cumulative incidence of overall survival  [Time Frame: 1 year]  9.Failure free survival  The cumulative incidence of failure free survival  [Time Frame: 1 year]  **Safety Analysis:**  Safety and tolerability will be assessed by incidence and severity of AEs and changes from baseline of all relevant parameters, including laboratory test values, physical examination, vital signs, and ECOG performance scores. With the exception of hematologic AEs, all AEs are graded according to CTCAE version 4.0. All subjects will be monitored for AEs within 1 year post-transplantation. |
|  |  |

**Abbreviations**

| AEs | Adverse events |
| --- | --- |
| allo-HSCT | Allogeneic hematopoietic stem cell transplantation |
| aGvHD | Acute graft versus host disease |
| ALT | Alanine aminotransferase |
| ANCs | Absolute neutrophil counts |
| AST | Aspartate aminotransferase |
| BM | Bone marrow |
| BSA | Body surface area |
| CIR | Cumulative incidences of relapse |
| CR | Complete remission |
| CRFs | Case Report Forms |
| CTCAE | Common Terminology Criteria for Adverse Events |
| CMV | Cytomegalovirus |
| cGvHD | Chronic graft versus host disease |
| DSMC | Data and safety monitoring committee |
| ECOG | Eastern Cooperative Oncology Group |
| FFS | Failure free survival |
| IRB | Institution review board |
| MP | Methylprednisolone |
| MTX | Methotrexate |
| NRM | Non-relapse mortality |
| OS | Overall survival |
| PLT | Platelets |
| SAEs | Serious adverse events |
| TBL | Total bilirubin |
| TPO | Thrombopoietin |
| ULN | Upper limits of normal |

# 1. Introduction

# Acute graft-versus-host disease(aGvHD) is a common complication and a major cause of morbidity and even mortality after allogeneic hematopoietic stem cell transplantation(allo-HSCT). Glucorticosteroids(GCs) remain to be the standard first-line therapy for aGvHD despite that 35-50% of aGvHD patients have no response to GCs. Moreover, no efficacy in response rate was observed in previous reports on combining GCs with other immunosuppressive agents including mycophenolate mofetil(MMF), antithymocyteglobulin(ATG), anti- interleukin-2 receptor antibody, infliximab, or itacitinib as the first-line therapy for aGvHD patients. A meta-analysis of such RCTs comparing various combined front-line therapies with steroids alone even revealed significantly inferior 100-d survival in the combination arm (relative risk 0.83, *P* = 0.004). Thus, the alternative first-line combination therapy by adding novel forms of immunomodulation against non-immune-related pathways is urgently to be explored.

Rather than the above mentioned generic immunosuppression, other means based on the different pathogenesis of aGvHD may enhance the efficacy of steroids. As a common immunosuppressive agent for GvHD, the effect of methotrexate (MTX) on metabolic checkpoints has been reported. Whether GCs combined with MTX could ameliorate aGvHD and synergistically improve T cell function needs to be investigated. Our group previously reported that, intravenous MTX at a dose of 10 mg or oral MTX at a dose of 15 mg combined with a low dose of 0.5mg/kg/d methylprednisolone (MP) yielded an overall treatment response in 26 out of 32 aGVHD patients (81%). Although this trial included a prospective cohort, there are some limitations with the pilot study. First, the data of 0.5mg/kg/d MP administration are difficult to be compared with results from standard dose of 1-2mg/kg/d MP used for first-line treatment of aGvHD. Second, the treatment response was evaluated until maximal response was achieved rather than a pre-defined fixed time point as suggested by standardized terminology and guidance for initial treatment response assessment. Considering these limitations, prospective studies with standard-dose MP plus lower-dose MTX and standardized assessment for initial treatment response are needed to challenge the standard first-line treatment for aGVHD of systemic steroids.

Recently, lower dose of 5mg/m^2^ instead of standard-dose of 10-15mg/m^2^ MTX was used either as prophylaxis or salvage therapy for aGvHD showing better safety control without compromising efficacy. Thus, a prospective, multicenter, randomized, controlled clinical trial is designed to identify the efficacy and safety of MTX combined corticosteroid as first-line therapy for aGvHD after allo-HSCT.

# 2. Study objectives

## 2.1 Primary Objective

The primary objective of this study is to compare the overall response rate (ORR) for aGvHD treatment at 10 days after treatment. Overall response rate is defined as the proportion of patients demonstrating a complete response or partial response without requirement for additional systemic therapies for an earlier progression, mixed response or non-response.

## 2.2 Secondary Objectives

The secondary objectives of this study are to compare ORR for aGvHD treatment at 28 days and 42 days after treatment, the cumulative incidences of cGvHD, leukemia relapse (CIR), non-relapse mortality (NRM), leukemia-free survival (LFS), overall survival (OS), and adverse effects (AEs) in aGvHD patients who receive allo-HSCT.

1. Overall response rate (ORR) for aGVHD treatment at 28 days after treatment

[Time Frame: 28 days]

2. Overall response rate (ORR) for aGVHD treatment at 42 days after treatment

[Time Frame: 42 days]

3. Number of participants with treatment-related adverse events as assessed by CTCAE v4.0

Data collection including questionnaires at individual and group visits and physician interviews at individual visits will be used to assess participants for treatment-related adverse events.

[Time Frame: 42 days]

4. cGVHD

The cumulative incidence of chronic GVHD

[Time Frame: 1 years]

5. Infection

The cumulative incidence of severe infection

[Time Frame: 1 year]

6. Relapse

The cumulative incidence of relapse

[Time Frame: 1 year]

7. Non-relapse mortality

The cumulative incidence of non-relapse mortality

[Time Frame: 1 year]

8. Overall survival

The cumulative incidence of overall survival

[Time Frame: 1 year]

9. Failure free survival

The cumulative incidence of failure free survival

[Time Frame: 1 year]

# 3. Study Design

This is a prospective, open-label, randomized, phase 3 study comparing MTX plus corticosteroid with corticosteroid alone for treating patients with acute graft versus host disease post allo-HSCT. Approximately 156 subjects will be randomized in a 1:1 ratio to receive MTX plus corticosteroid (78 subjects) or corticosteroid (78 subjects) for treating aGvHD post-transplantation. The computer-generated randomization codes were sent to the IWRS vendor to implement the randomization, and implemented through an interactive web-based response system.

**Study Group:** Methylprednisolone 1 mg/kg/day was given for 10 days and then gradually reduce the dose according to patient's response MTX (5mg/m^2/day, Maximum dose 10mg/day) was given on days 1, 3, and 8, and repeated weekly until aGVHD was CR.

**Control Group:** Methylprednisolone 1 mg/kg/day was given for 10 days and then gradually reduce the dose according to patient's response.

Subjects with grade I-IV aGvHD will be screened for eligibility. Medical history evaluation, vital sign, physical examination, Eastern Cooperative Oncology Group (ECOG) performance status, blood and urine sampling for laboratory tests, electrocardiogram, as well as chest imaging examination will be performed to determine study eligibility. Eligible subjects will be randomized in a 1:1 ratio to receive MTX plus corticosteroid versus corticosteroid as first-line therapy for aGvHD. Randomization is performed with randomization codes generated by a computer-generated randomization system.

Based on the randomization and assignment, the subjects will receive MTX plus corticosteroid or corticosteroid as first-line therapy for aGvHD (Figure 1). All subjects will be followed for safety and tolerability within 1-year post-transplantation. With the exception of hematologic AEs, all AEs are graded according to CTCAE version 4.0.

**Figure 1 Study Schema**

**
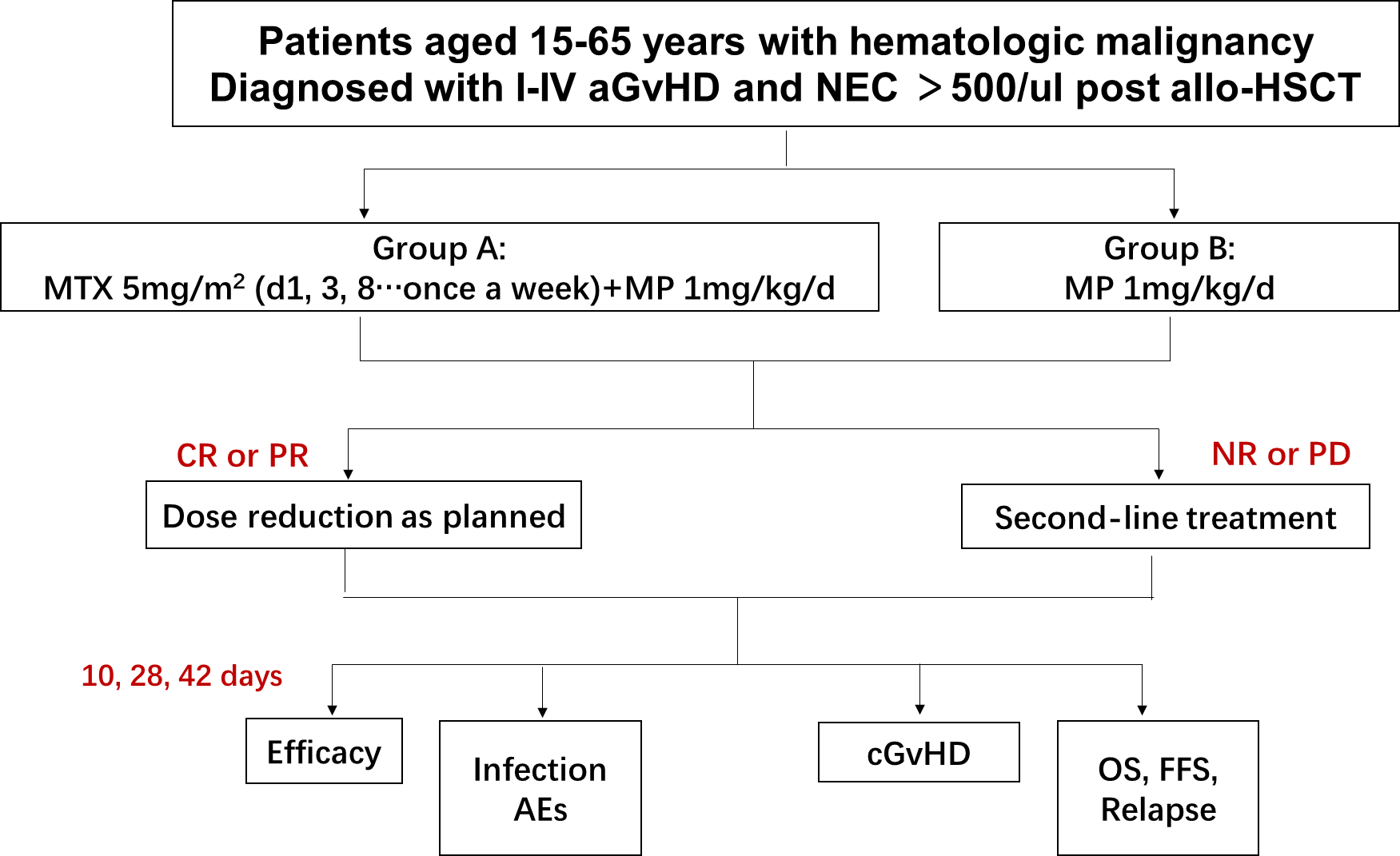
**

# 4. Subject Selection Criteria

## 4.1 Subject Selection Criteria

### 4.1.1 Number of Subjects

Approximately 156 subjects will be randomized to Study or control group (78 subjects in Study group and 78 in control group).

### 4.1.2 Inclusion Criteria

Subjects eligible for enrolment in this study must meet all of the following criteria:

1. Aged 15-65 years old;

2. Patients who are fully informed and sign informed consent by themselves or their guardians;

3. Patients receiving allogeneic hematopoietic stem cell transplantation;

4. Patients with acute graft-versus-host disease of grade I-IV were diagnosed after transplantation;

5. Patients with stable engraftment of myeloid and platelet;

6. ECOG score ≤3.

### 4.1.3 Exclusion Criteria

Subjects meeting any of the following criteria are ineligible for this study:

1. Patients with more than one HSCT;

2. Patients with severe brain, heart, kidney or liver dysfunction unrelated to graft-versus host disease;

3. Patients with uncontrollable active infection;

4. Patients with recurrence of hematologic malignant disease;

5. Expected survival is less than 3 months;

6. Patients who have histories of severe allergic reactions;

7. Pregnant or lactating women;

8. The researcher judges that there are other factors that are not suitable for participating.

## 4.2. Withdrawal Criteria

Subjects are free to withdraw consent and discontinue participation in the study at any time and without prejudice to future treatment. A subject's participation in the study may be discontinued at any time at the investigator's discretion. Justifiable reasons for a subject to be withdrawn from the study include:

1. Inability to fully comply with the study protocol

2. Initiation of aGvHD treatment of other alternative choices except MTX or MP

3. Unacceptable toxicity

4. Best interest of the subject based upon the investigator’s discretion

5. At the request of the study subject at any time and for any reason

Subjects will be followed up unless the informed consent is withdrawn. The reason for withdrawal from study participation and the date must be documented in the case report form (CRF). The investigator must complete the last visit, including vital signs, physical examination, laboratory tests, disease status and AE assessment, all of which must be documented in the CRF.

# 5. Study Procedures

## 5.1 Screening

Subjects with aGvHD post allo-HSCT will be screened for eligibility. Medical history evaluation, vital sign, physical examination, ECOG performance status, blood and urine sampling for laboratory tests, electrocardiogram, as well as chest imaging examination will be performed to determine study eligibility.

## 5.2 Treatment Allocation and Blinding

This is an open-label study. Neither subjects nor investigators will be blinded to treatment. Upon completion of all the required screening assessments, eligible subjects will be randomized at 1:1 ratio to receive MTX plus methylprednisolone or methylprednisolone. The computer-generated randomization codes were sent to the IWRS vendor to implement the randomization, and implemented through an interactive web-based response system. Centralized randomization numbers within each stratum were created for treatment assignment, and site staff were instructed to contact the IWRS to obtain the patient identification numbers and initial study drug assignment. The next assignment in the sequence was concealed. The investigators or subjects were not masked to assignment. The outcome assessments and data analysis were undertaken in a masked pattern.

## 5.3 Study Treatment

## 5.3.1 Study Group (MTX plus methylprednisolone)

After enrollment, patients in the Study group were scheduled for MTX plus methylprednisolone.

1. **Drug administration:** Methylprednisolone 1mg/kg/day (or equivalent amount of prednisone), and MTX 5mg/m^2^/once (the maximum dose 10mg/once), intravenous infusion.

② **Dosage frequency:** Methylprednisolone 1mg/kg/day, divided twice; MTX D1, D3, D8, then once a week.

③ **Administration course:** Patients were scheduled to receive at least two doses (number of MTX administrations) for evaluation of the drug’s efficacy. If patients responded and were able to tolerate the toxicity, additional doses were used for consolidation and the scheduled maximal doses were capped at 6.

④ **Dosage reduction:** The protocol suggested a tapering steroid regimen (the programmed dose of methylprednisolone (MP) was: days 1-7, 1 mg/kg/d; days 8-14, 0.8 mg/kg/d; days 15-21, 0.5mg/kg/d and the dose was reduced by half after 5-7 days until it was stopped), but it was not mandated. In general, the taper could not commence sooner than 7 days after randomization.

⑤ **Second-line treatment**: Patients were observed for 10 days and switched to the second-line treatment if there was no response to the initial therapy. The second-line treatment was given to patients at 5-7 days with progression of aGVHD or 10 days with no improvement after initial therapy, not including patients who responded to MTX/MP at first and then flared. The typical sequence of secondary therapy was to add a nonglucocorticoid agent because further escalation of glucocorticoid doses has not been the standard practice at study sites. The second-line treatments followed the local institutional practice which include basiliximab (Novartis Pharma AG, Basel, Switzerland) at 20 mg/d on days 1, 3, 8, and weekly afterwards for as long as was clinically indicate, ruxolitinib or MMF. For GVHD flares occurring during the corticosteroid taper period, the corticosteroid dose could be re-escalated at the discretion of the investigator and was not considered treatment failure as long as the escalated dose did not exceed the 2mg/kg MP.

## 5.3.2 Control Group

① **Drug administration:** Methylprednisolone 1mg/kg/d (or equivalent amount of prednisone), intravenous infusion;

1. **Dosage frequency:** Methylprednisolone 1mg/kg/d, divided twice;

③ **Dosage reduction:** The protocol suggested a tapering steroid regimen (the programmed dose of methylprednisolone (MP) was: days 1-7, 1 mg/kg/d; days 8-14, 0.8 mg/kg/d; days 15-21, 0.5mg/kg/d and the dose was reduced by half after 5-7 days until it was stopped), but it was not mandated. In general, the taper could not commence sooner than 7 days after randomization.

④ **Second-line treatment:**  Patients were observed for 10 days and switched to the second-line treatment if there was no response to the initial therapy. The second-line treatment was given to patients at 5-7 days with progression of aGVHD or 10 days with no improvement after initial therapy, not including patients who responded to MTX/MP at first and then flared. The typical sequence of secondary therapy was to add a nonglucocorticoid agent because further escalation of glucocorticoid doses has not been the standard practice at study sites. The second-line treatments followed the local institutional practice which include basiliximab (Novartis Pharma AG, Basel, Switzerland) at 20 mg/d on days 1, 3, 8, and weekly afterwards for as long as was clinically indicate, ruxolitinib or MMF. For GVHD flares occurring during the corticosteroid taper period, the corticosteroid dose could be re-escalated at the discretion of the investigator and was not considered treatment failure as long as the escalated dose did not exceed the 2mg/kg MP.

## 5.4 Follow-up

All subjects will be followed for evaluation, safety and tolerability within 1 year post-transplantation. With the exception of hematologic AEs, all AEs are graded according to CTCAE version 4.0.

# 6. Efficacy Assessments

## 6.1 Definitions

- Prior to initiation of the treatment, patients underwent a thorough evaluation to ascertain the severity and extent of their GVHD, including a physical examination, laboratory evaluations and a consultation without the tissue biopsy results. Each organ (skin, liver, gut) was staged 1 through 4 for acute GVHD according to modified criteria based on the schema of the Mount Sinai Acute GVHD International Consortium (MAGIC), and patients were also assigned a grade of acute GVHD (I through IV) based on overall severity. Minnesota GVHD risk status was also evaluated.
- Chronic GvHD was defined and graded according to the National Institute of Health criteria: [Biol Blood Marrow Transplant,2005,11: 945] that is, mild cGvHD reflects the involvement of no more than 1 or 2 organs/sites (except for lung) with a maximum score of 1; moderate cGvHD involves at least 1 organ/site with a score of 2 or ≥3 organs/sites with a score of 1 (or lung score 1); and severe cGvHD is diagnosed when a score of 3 is given to any organ (or lung score 2). The diagnosis is mainly based on clinical manifestations.
- Overall response rate (aGvHD) is defined as the proportion of patients demonstrating a complete response or partial response without requirement for additional systemic therapies for an earlier progression, mixed response or non-response.
- Neutrophil engraftment was defined as maintenance of an absolute neutrophil count above 0.5×109/L for three consecutive days after the neutrophil nadir.
- Platelet engraftment was defined as the first of 7 consecutive days during which the nontransfused platelet count was at least 20×109/L.
- CMV or EBV reactivation was defined as a CMV DNA viral load ＞600 copies/mL or an EBV DNA viral load ＞500 copies/mL at any time after HSCT for at least one measurement.
- Relapse is defined as reappearance of leukemic blasts in the peripheral blood or ≥ 5% blasts in the BM as pirate or biopsy not attributable to any other cause or reappearance or new appearance of extramedullary leukemia.
  - CR is defined as BM blasts <5%; absence of circulating blasts and blasts with Auer rods; absence of extramedullary disease; ANC ≥1.0×10^9^/L and PLT ≥100×10^9^/L.

## 6.2 Primary Efficacy Endpoint

- Overall response rate (ORR) for aGvHD treatment after treatment

Overall response rate is defined as the proportion of patients demonstrating a complete response or partial response without requirement for additional systemic therapies for an earlier progression, mixed response or non-response.

## 6.3 Secondary Efficacy Endpoints

- - To compare the cumulative incidences of overall response rate (ORR) for aGvHD treatment at 28 days, 42 days after treatment, number of participants with treatment-related adverse events as assessed by CTCAE v4.0, cGvHD, infection, relapse, non-relapse mortality, overall survival and failure-free survival between the two groups.

## 6.4 Schedule and methods of Efficacy Assessments

- The occurrence and treatment of acute and chronic GvHD after transplantation
- Basic items: routine blood examination (twice a week), liver, kidney, coagulation function examination, CMV and EBV (weekly)
- Bone marrow morphology, residual leukemia (MRD) and related genes monitored at 1, 2, 3, 4.5, 6, 9 and 12 months post transplantation
- Immune reconstitution: lymphocyte subsets (CD4+, CD4+, CD4+, CD4+, CD8+, CD19+, etc.) and immunoglobulin levels

# 7. Safety Evaluation

The safety-evaluable population included all patients who received at least one dose of study drug. Safety and tolerability will be assessed with vital signs, physical examination, clinical symptoms, and clinical laboratory evaluations (hematology, serum chemistry, urinalysis, electrocardiogram, and chest imaging examination). With the exception of hematologic AEs, all AEs will be evaluated within 60 days post-transplantation according to CTCAE version 4.0.

### 7.1 Medical History

Each subject's medical history must be obtained at screening. Information on any prior or existing medical conditions will be recorded on the appropriate CRF.

### 7.2 Vital Signs and Physical Examination

Vital signs and results of physical examination must be documented before randomization, once a week for the first month after enrollment, once every two weeks from engraftment until 60 days post-transplantation. The next 8 items must be performed:

⚫ Physical examination

⚫ Heart rate

⚫ Blood pressure

⚫ Body temperature

⚫ Rate of respiration

⚫ Body weight

⚫ ECOG performance status

⚫ Signs of infection

### 7.3 Clinical Symptoms

During the study, the patients' clinical symptoms must be documented. The clinical symptoms may be associated with occurrence of aGvHD and the administration of MTX reported previously.

### 7.4 Clinical Laboratory Evaluations

Before initiation of the study, the monitors will document the normal range of each test in every involved laboratory. During the study, the next items must be performed:

⚫ Routine blood: white cell counts, neutrophil cell counts, hemoglobin, and platelet counts

⚫ Hepatic function: total bilirubin (both direct bilirubin and indirect bilirubin must be documented when the total bilirubin elevates), ALT, AST, lactic dehydrogenase, alkaline phosphatase, albumin and total protein

- Renal function: serum creatinine, urea nitrogen and uric acid
- Other biochemical indicators: amylase and lipase
- Electrolytes: sodium, potassium, calcium and magnesium
- Coagulation function: prothrombin time, prothrombin time-international normalized ratio, activated partial thromboplastin time and fibrinogen
- Urinalysis: protein, glucose and erythrocyte
- Electrocardiogram
- Chest imaging examination

## 8 Adverse Events and Serious Adverse Events (SAEs)

The investigator is responsible for detecting, documenting and reporting events that meet the definition of an AE or SAE.

### 8.1. Definitions

## 8.1.1 Adverse Events

An AE is any untoward medical occurrence in a subject of a clinical investigation, which does not necessarily have a causal relationship to the medicinal product. Therefore, an AE can be any unfavorable and unintended sign, including an abnormal laboratory finding, symptom, or disease (new or exacerbated), whether or not it is considered to be related to the product. This definition includes any newly occurring event or previous condition that has increased in severity or frequency since the administration of the product. However, transplantation related mortality should not be recorded as AEs.

## 8.1.2 Serious Adverse Events

A serious adverse event is any untoward medical occurrence that, at any dose:

- - Results in death
  - Is life-threatening
  - Requires hospitalization or prolongation of existing hospitalization - ie, the AE requires at least a 24-hour inpatient hospitalization or prolongs a hospitalization beyond the expected length of stay.

Hospitalization or prolongation of existing hospitalization for social reasons will not be reported as an SAE.

- - Results in disability/incapacity
  - Congenital anomaly/ birth defect
  - Important medical event

Medical or scientific judgment should be exercised in deciding whether SAE reporting is appropriate in other situations. An important medical event is an event that may not result in death, be life-threatening, or require hospitalization, but is clearly of major clinical significance. The AE may jeopardize the subject or require intervention to prevent a serious outcome.

## 8.2 Assessment of Severity

all AEs are graded according to CTCAE version 4.0. When CTCAE version 4.0 criteria do not apply, severity will be defined according to the following criteria:

| **Severity** | **Description** |
| --- | --- |
| Grade 1- Mild | Asymptomatic or mild symptoms; clinical or diagnostic observations only; intervention not indicated |
| Grade 2- Moderate | Minimal, local or noninvasive intervention indicated; limiting age-appropriate instrumental activities of daily living (ADL) |
| Grade 3- Severe | Medically significant but not immediately life threatening; hospitalization or prolongation of hospitalization indicated; disabling; limiting self-case ADL |
| Grade 4- Life-threatening | Life-threatening consequences; urgent intervention indicated |
| Grade 5- Death | Death |

## 8.3 Assessment of Causality

The investigator must determine the relationship of each AE and SAE to study treatment. Relationship of an AE or SAE to study treatment will be defined according to the following criteria:

- - Definite: There is a clear temporal relationship to study treatment, with no other possible cause.
  - Possible: A temporal relationship to study treatment is not clear, and alternative etiologies are possible.
  - Not related: There is no temporal relationship to study treatment, and/or there is evidence of an alternative cause such as a concurrent medication or illness.

## 8.4 Recording and Reporting AEs and SAEs

All AEs and SAEs must be recorded in the appropriate CRF, whether or not they are associated to be causally related to study treatment. Each SAE must be reported promptly on the Serious Adverse Event Report Form, and submitted to the Independent Ethics Committee within 24 hours by the investigator. The information recorded on the Serious Adverse Event Report Form will include at least the following: subject number, identity of the event, study drug name and dose, investigator's assessment of the event's severity and relationship to study treatment, and investigator's name and signature. Clinical monitors must collect and verify detailed information of AEs and SAEs when examining original medical records. All AEs and SAEs should be followed up until resolved.

# 9. Rules of Withdrawal

## 9.1. Subjects Withdraw from the Study

Subjects can withdraw from the study at any time for any reason without impact on the investigator’s right to treat disease for subjects. Based upon the interest of subjects, the investigator has the right to request subjects to withdraw from the study for any reason including concomitant disease, AEs or treatment failure. The core group of clinical study reserves the right to request subjects to withdraw from the study for deviation(s) from the protocol, administrative reasons, or other effective or ethical reasons.

The last assessment for subjects must be performed and documented in the CRF regardless of the time and reason for withdrawal. The reason for withdrawal from study participation must be documented in the CRF. All documents related to subjects should be completed. Despite withdrawal from the study, those subjects should be followed up and documented about their diseases until withdrawal of informed consents.

For subjects who withdraw from the study due to concomitant diseases or AEs, the details must be documented in the CRF with other appropriate and valuable data attached.

## 9.2. Premature Termination of the Study

Reasons for premature termination of the study include external events, repetition of SAEs, growing incidence of treatment-related death and slow enrolment in the study. All subjects will be informed of premature termination of the study by written consents. Any subjects who decide to discontinue participating in the study must report to the principal investigator.

# 10. Rules of Follow-Up

## 10.1 Follow-up Period

Starting from randomization.

## 10.2 Visit Scheduling

Patients underwent acute GVHD assessments by their treating physicians at screening; every other day for the first 10 days on treatment; at the end of treatment; on days 14, 28, and 42; every 28 days (range 25–31) during re-treatment, if applicable; at safety follow-up (42 days after the end of treatment); and at GVHD follow-up (every 14 days for patients who completed treatment or discontinued early for reasons other than GVHD progression). Prior to initiation of the treatment, patients underwent a thorough evaluation to ascertain the severity and extent of their GVHD, including a physical examination, laboratory evaluations and a consultation without the tissue biopsy results.

## 10. 3 Contents

Prior to initiation of the treatment, patients underwent a thorough evaluation to ascertain the severity and extent of their GVHD, including a physical examination, laboratory evaluations and a consultation without the tissue biopsy results.

The contents of every follow-up visit include complaints of subjects, vital signs, physical examination, clinical symptoms and clinical laboratory evaluations (hematology, serum chemistry, urinalysis, electrocardiogram, chest imaging examination, and BM assessment). All of the results must be documented in the original medical record.

# 11. Data Analysis and Statistical Considerations

## 11.1. Hypotheses

The primary endpoint is the overall response rate of aGvHD post-transplantation. The null and alternative hypotheses are designed with the goal of demonstrating the superiority of MTX plus MP over MP with respect to treating aGvHD. Superiority will be determined using the following hypothesis:

H0: ORR with MTX plus MP ≥ ORR with MP alone

H1: ORR with MTX plus MP＜ORR with MP alone

## 11.2 Study Design Considerations

This trial was designed to test the hypothesis that MTX plus methylprednisolone was superior to methylprednisolone in the treatment of aGvHD post transplantation.

A sample size of 142 patients was calculated using continuity correction to allow for the detection of an absolute improvement in ORR at day 10 of 20% (ie, 90% for MTX vs 70% for control) with 80% statistical power (one-sided alpha 0.025). The assumed ORR of 70% was based on a grade ≤II:grade≥III ratio of 0.90:0.10, with a stratum-specific response rate of 75% and 30%, respectively. After adjusting for a 10% dropout, the total planned sample size was 156 patients. The sample size calculation was performed using PASS software(version 11.0).

### 11.2.1 Sample Size Assumptions

The sample size calculation is based on the primary endpoint, the ORR of aGvHD at 10 days post treatment, with the following assumptions:

- - - - ORR of aGvHD in the control group: 70%
      - ORR of aGvHD in the study group: 90%
      - a 1:1 randomization scheme
      - a 5% one-tailed risk of erroneously claiming a difference in the presence of no true underlying difference by z-test with pooled variance
      - an 80% chance of successfully declaring a difference in the presence of a true underlying difference (power)
      - 10% percent of cases drop

Under the above assumptions, a total sample size of 156 subjects is required (78 in study group and 78 in control group).

### 11.2.2 Primary Efficacy Endpoint

The primary efficacy endpoint is the Overall response rate (ORR) for aGvHD treatment at 10 days after treatment.

### 11.2.3 Secondary Efficacy Endpoints

The secondary efficacy endpoints include overall response rate (ORR) for aGvHD treatment at 28 days, 42 days after treatment, number of participants with treatment-related adverse events as assessed by CTCAE v4.0, cGvHD, infection, relapse, non-relapse mortality, overall survival and failure-free survival between the two groups.

## 11.3 Data Analysis Considerations

### 11.3.1 Analysis Population

The primary population will be the intent-to-treat (ITT) population, which is defined as all subjects randomized to the two groups. This ITT population will be the basis for the analysis of efficacy endpoints in this study. The safety-evaluable population included all patients who received at least one dose of study drug.

### 11.3.2 Analysis Plan

### 11.3.2.1 Baseline Data

Baseline characteristics will be summarized and described in a frequency list.

### 11.3.2.2 Analysis of Efficacy

The primary endpoint (day 10 ORR) was assessed via Cochran-Mantel-Haenszel test with normal approximation, stratified by GVHD grading. The time course for aGVHD response was estimated using the method of Kaplan and Meier. Cumulative incidences of malignancy relapse, NRM, and chronic GVHD were calculated by accounting for competing risks using the Fine and Gray model. OS and FFS were estimated by the Kaplan-Meier method and compared by the log-rank test. The corresponding hazard ratio(HR) and 95% CI, were estimated using the Cox proportional hazards model. All variables in Table 1 were included in the univariable analysis. Only variables with P<.15 were included in the multivariable analysis. Subgroup analyses for the primary endpoint included evaluation of age, sex, human leukocyte antigen or donor source category, stem cell source category，baseline acute GVHD grade and risk status, and organ involvement at baseline. Summary statistics and 95% CIs are reported for study endpoints, as applicable. A post-hoc analysis was done to assess complete response rates at day 10. All reported p values from the primary efficacy analyses are two-sided.

The efficacy-evaluable population included all randomized patients (full analysis set) and was used to summarize baseline characteristics, patient disposition, and analyses of all efficacy data according to the intention-to-treat principle. The SPSS 19.0 (Mathsoft, Seattle, WA, USA) and R version 3.4.4 (The R Foundation for Statistical Computing) were used for data analyses.

### 11.3.2.3 Analysis of Safety

The safety-evaluable population included all patients who received at least one dose of study drug. No statistical comparisons were done for safety endpoints. Safety and tolerability will be assessed by incidence and severity of AEs and changes from baseline of all relevant parameters, including laboratory test values, physical examination, vital signs, and ECOG performance scores. The definition of AEs has been detailed in previous section. With the exception of hematologic AEs, all AEs are graded according to CTCAE version 4.0. All subjects will be monitored for AEs within 60 days post-transplantation. Categorical data will be summarized by proportion of total subjects. Quantitative data will be described using arithmetic average or median for central tendency and standard deviation or interquartile range for distribution range.

# 12. Materials for the Study

All materials provided to study sites and investigators are as follows:

- The study protocol
- Informed consent
- CRF

# 13. Ethical Considerations

## 13.1 Responsibility of Investigators

The investigators have the responsibility for guarantee of the clinical study’s compliance with the protocol, Chinese good clinical practice (GCP) guidelines and applicable laws and regulations.

## 13.2 Informed Consent Process

Prior to participation in the study, subjects must be informed about objectives, methods, possible benefits, potential risks and possible discomforts of the study by investigators. They also should be informed that participation in the study would be voluntary, they can withdraw from the study at any time, there is no impact on the treatment of the disease whether they take part in the study and their privacy will be protected.

Subjects or their legally acceptable representative should have enough time to read the inform consent and raise queries. Written informed consent must be obtained from each subject, or their legally acceptable representative.

## 13.3 Good Clinical Practice

This study will be conducted in accordance with the Declaration of Helsinki and Chinese GCP. The study will be conducted only if it is approved by the ethical review committee of the principal study site. The investigators will guarantee that the study will be conducted in accordance with applicable laws and regulations, scientific and ethical principles of the People’s Republic of China. If the protocol needs revision during the study, the revised version must be reapproved by the ethical review committee of the principal study site. If new data related to study treatment are discovered, the informed consent must be revised and the revision must be reapproved by the ethical review committee of the principal study site and subjects.

## 13.4 Protection of Subjects’ Personal Data

Data collected in the study are limited to the efficacy and safety related to study treatment. Data will be collected and used in accordance with applicable laws and regulations.

# 14. Administrative Requirements

Neither the investigator nor the applicant can revise the protocol without agreement of the opposite side. All revisions of the protocol must be released by the applicant institution. To insure the integrity, accuracy and reliability of the data, relevant results of examination and treatment must be documented in original medical record and CRF. Independent clinical monitoring is performed regularly by a panel of qualified and experienced study investigators composed of hematologists who are blinded as to the treatment assignments.

# 15 Appendices

## 15.1 Appendix 1 Diagnosis and Classification of aGVHD and cGVHD

**Grading of aGVHD**

Each organ (skin, liver, gut) was staged 1 through 4 for acute GVHD according to modified criteria based on the schema of the Mount Sinai Acute GVHD International Consortium (MAGIC), and patients were also assigned a grade of acute GVHD (I through IV) based on overall severity.

| **Stage** | **Skin (active erythema only)** | **Liver (bilirubin)** | **Upper GI** | **Lower GI (stool output/day)** |
| --- | --- | --- | --- | --- |
| 0 | No active (erythematous) GVHD rash | ＜2mg/dl | No or intermittent nausea, vomiting or anorexia | Adult: ＜500ml/day or ＜3 episodes/day  Child: ＜10ml/kg/day or ＜4 episodes/day |
| 1 | Maculopapular rash ＜25% BSA | 2-3 mg/dl | Persistent nausea, vomiting or anorexia | Adult: 500-999ml/day or 3-4 episodes/day  Child: 10-19.9ml/kg/day or ＜4-6 episodes/day |
| 2 | Maculopapular rash 25%-50% BSA | 3.1-6 mg/dl | - | Adult: 1000-1500ml/day or 5-7 episodes/day  Child: 20-30ml/kg/day or ＜7-10 episodes/day |
| 3 | Maculopapular rash ＞50% BSA | 6.1-15 mg/dl | - | Adult: ＞1500ml/day or ＞7 episodes/day  Child: ＞30ml/kg/day or ＞10 episodes/day |
| 4 | Generalized erythrodema (＞50% BSA) plus bullous formation and desquamation ＞5% BSA | ＞15 mg/dl | - | Severe abdominal pain with or without ileus, or grossly bloody stool (regardless of stool volume). |

**Overall clinical grade (based upon most severe target organ involvement):**

Grade 0: No stage 1–4 of any organ

Grade I: Stage 1–2 skin without liver, upper GI or lower GI involvement

Grade II: Stage 3 rash and/or stage 1 liver and/or stage 1 upper GI and/or stage 1 lower GI

Grade III: Stage 2–3 liver and/or stage 2–3 lower GI, with stage 0–3 skin and/or stage 0–1 upper GI

Grade IV: Stage 4 skin, liver or lower GI involvement, with stage 0–1 upper GI

International, multicenter standardization of acute graftversus-host disease clinical data collection: a report from the Mount Sinai Acute GVHD International Consortium. *Biol Blood Marrow Transplant*: J Am Soc Blood Marrow Transplant.2016.

**Minnesota GVHD risk status**

| GVHD Risk Score | One Organ | Two Organs | Three Organs |
| --- | --- | --- | --- |
| Standard-risk | Stage 1-3 Skin  Stage 1-2 GI | Stage 1-3 skin plus stage 1 GI  Stage 1-3 skin plus stage 1-4 liver | —  — |
| High-risk | Stage 4 Skin  Stage 3-4 GI  Stage 1-4 Liver | Stage 1-3 skin plus stage 2 GI  Stage 1-2 lower GI plus stage 1-3 liver  Stage 3-4 GI plus stage 1-3 skin  Stage 3-4 GI plus stage 1-4 liver | Stage 1-3 skin plus stage 1-2 GI plus  stage 1-3 liver  Stage 1-3 skin plus stage 3-4 GI plus  stage 1-4 liver |

**GVHD Risk Definition by Organ Stage at Onset.**

A refined risk score for acute graft-versus-host disease that predicts response to initial therapy, survival, and transplant-related mortality. *Biol Blood Marrow Transplant* 2015.

**Grading of cGVHD**

| NIH Global Severity of chronic GVHD |
| --- |
| Mild chronic GVHD |
| 1 or 2 Organs involved with no more than score 1 plus Lung score 0 |
| Moderate chronic GVHD |
| 3 or More organs involved with no more than score 1 |
| OR |
| At least 1 organ (not lung) with a score of 2 |
| OR |
| Lung score 1 |
| Severe chronic GVHD |
| At least 1 organ with a score of 3 |
| OR |
| Lung score of 2 or 3 |
| Key points:  In skin: higher of the 2 scores to be used for calculating global severity.  In lung: FEV1 is used instead of clinical score for calculating global severity.  If the entire abnormality in an organ is noted to be unequivocally explained by a non-GVHD documented cause, that organ is not included for calculation of the global severity.  If the abnormality in an organ is attributed to multifactorial causes (GVHD plus other causes), the scored organ will be used for calculation of the global severity regardless of the contributing causes (no downgrading of organ severity score). |

National Institutes of Health Consensus Development Project on Criteria for Clinical Trials in Chronic Graft-versus-Host Disease: I. The 2014 Diagnosis and Staging Working Group report. *Biol Blood Marrow Transplant.* 2015.


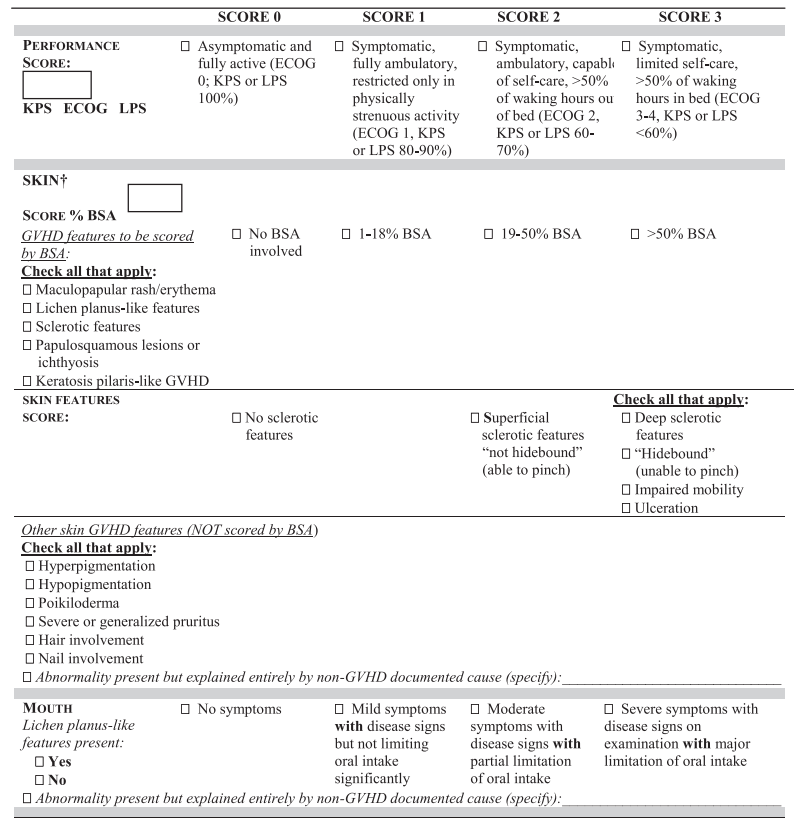


Figure1. Organ scoring of chronic GVHD. ECOG indicates Eastern Cooperative Oncology Group; KPS, Karnofsky Performance Status; LPS, Lansky Performance Status; BSA, body surface area; ADL, activities of daily living; LFTs, liver function tests; AP, alkaline phosphatase; ALT, alanine aminotransferase; ULN, normal upper limit. *Weight loss within 3 months. †Skin scoring should use both percentage of BSA involved by disease signs and the cutaneous features scales. When a discrepancy exists between the percentage of total body surface (BSA) score and the skin feature score, OR if superfificial sclerotic features are present (Score 2), but there is impaired mobility or ulceration (Score 3), the higher level should be used for the final skin scoring. **Lung scoring should be performed using both the symptoms and FEV1 scores whenever possible. FEV1 should be used in the final lung scoring where there is discrepancy between symptoms and FEV1 scores.


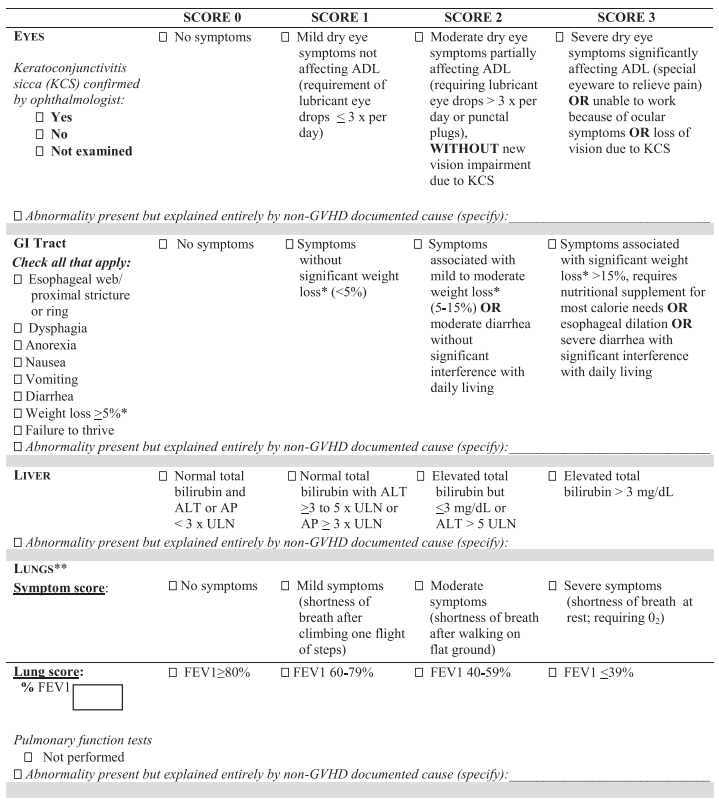


Figure 1. (continued).


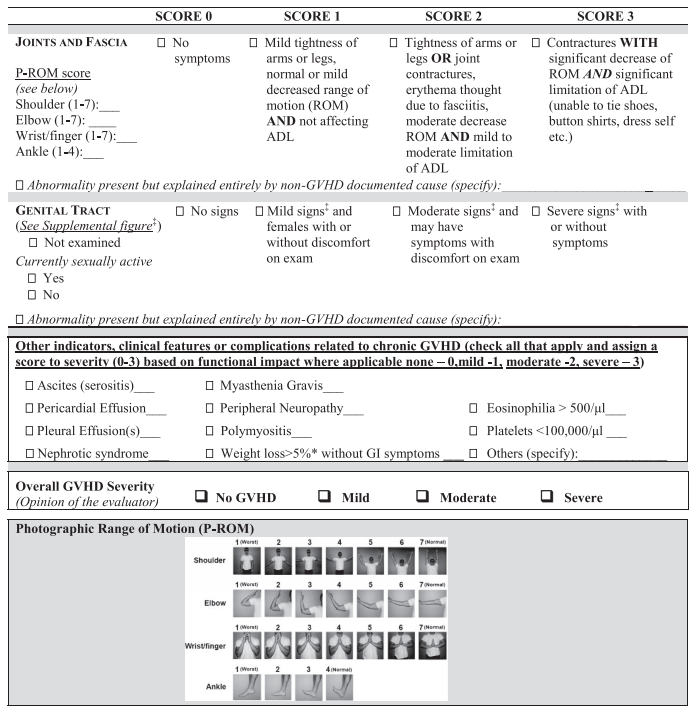


Figure 1. (continued).

National Institutes of Health Consensus Development Project on Criteria for Clinical Trials in Chronic Graft-versus-Host Disease: I. The 2014 Diagnosis and Staging Working Group report. *Biol Blood Marrow Transplant.* 2015.
